# Comparative evaluation of three commercial real-time PCR kits for direct detection of *Mycobacterium tuberculosis* complex and non-tuberculous mycobacteria from pulmonary and extrapulmonary clinical samples: experiences from a low-income and high-burden setting

**DOI:** 10.1128/spectrum.00158-26

**Published:** 2026-05-18

**Authors:** Ángel Sebastián Rodríguez-Pazmiño, Elsy Carvajal, Bernardo Castro-Rodriguez, Greta Franco-Sotomayor, Greta Cardenas-Franco, Sandra Uruchima, Solon Alberto Orlando, Miguel Angel Garcia Bereguiain

**Affiliations:** 1 One Health Research Group, Universidad de Las Americas 126422, Quito, Ecuador; 2 Instituto Nacional de Salud Pública e Investigación 370452, Guayaquil, Ecuador; 3 Universidad Católica Santiago de Guayaquil 27887, Guayaquil, Ecuador; 4 Universidad Ecotec 252896, Samborondón, Ecuador; Ascension St John Hospital, Detroit, Michigan, USA

**Keywords:** tuberculosis, *Mycobacterium tuberculosis* complex, non-tuberculous mycobacteria, molecular diagnostic, commercial PCR real-time

## Abstract

The accurate and timely detection of *Mycobacterium tuberculosis* complex (MTBC) and non-tuberculous mycobacteria (NTM) remains a diagnostic challenge, particularly in extrapulmonary samples and low-resource settings. This study aimed to evaluate and compare the clinical performance of three commercial real-time polymerase chain reaction (PCR) assays, VIASURE *M. tuberculosis complex +* non-tuberculous mycobacteria Real-Time PCR Detection Kit (“Viasure”), Mycobacterium Multiplex Nucleic Acid Diagnostic Kit (“Sansure”), and Anyplex MTB/NTMe Real-Time Detection (“Seegene”), against conventional diagnostic methods (smear microscopy and culture) for the detection of MTBC and NTM in pulmonary and extrapulmonary samples. We analyzed 407 clinical samples from patients with suspected tuberculosis (TB) or other mycobacterioses. All samples were tested by smear microscopy, mycobacterial culture, and three real-time PCR assays. First, we compared the kits against smear and culture separately (scheme 1). Second, we used a composite reference standard (CRS) that classified a sample as positive if any of the five tests were positive and evaluated smear, culture, and the three PCRs for MTBC and NTM (scheme 2). Third, we compared Viasure and Sansure to Seegene as a reference, excluding PCR positives with Ct ≥ 35 to focus on high-confidence results (scheme 3). Overall positivity was lowest for smear (14.8%; 41/278) and culture (32.7%; 48/147), intermediate for Sansure (18.7%; 52/278), and highest for Viasure (60.1%; 167/278) and Seegene (53.2%; 148/278), both in pulmonary and extrapulmonary samples. In scheme 1, using smear or culture as references, Viasure and Seegene showed high sensitivity but modest specificity, which is, in fact, attributable to the poorer clinical performance of the reference methods. Under the CRS in scheme 2, smear and culture detected only about 20% and 46% of CRS-positive cases, respectively, whereas Viasure and Seegene captured most MTBC infections (total MTBC sensitivities of 0.73 and 0.71, respectively). For both schemes 1 and 2, Sansure was clearly less sensitive than the other kits to detect MTBC. For NTM, Viasure markedly outperformed the other kits (total sensitivity 0.88; specificity 0.98), whereas Sansure and Seegene showed very low NTM sensitivity (0.19 and 0.13) despite near-perfect specificity. In scheme 3, restricting analyses to Ct < 35 and using Seegene as reference yielded very high MTBC agreement between kits: Viasure was more sensitive (0.97) but slightly less specific (0.89), while Sansure was less sensitive (0.84) but almost perfectly specific (0.99); NTM performance could not be reliably assessed in this scheme due to the scarcity of Seegene NTM positives after applying the Ct filter. Our findings show that Viasure and Seegene substantially outperform smear microscopy and culture for MTBC detection in both pulmonary and extrapulmonary specimens, with Viasure offering the best overall balance and clearly superior NTM detection. Sansure behaves as a very specific but under-sensitive option, contributing comparatively few additional diagnoses beyond conventional methods. In medium to high-burden settings where rapid and accurate differentiation of TB and NTM from clinical samples is crucial, these results support the use of highly sensitive PCR assays (particularly Viasure and Seegene) as central tools within diagnostic algorithms, complemented by culture for drug susceptibility testing and full species characterization.

**IMPORTANCE:**

In this study, we have carried out an extensive validation of the three commercial qPCR kits available in Ecuador for the diagnosis of *Mycobacterium tuberculosis* and non-tuberculous mycobacteria, comparing them with standard methods. This evaluation was made with an extensive population of more than 400 pulmonary and extrapulmonary clinical samples. We found that two of the commercial kits are equivalent in diagnostic performance and outperformed traditional methods for mycobacterial diagnosis, while the third kit had a very poor performance. To the best of our knowledge, for two of the three commercial kits included in the study, there are no publications evaluating their clinical performance. This study provides useful information for the clinical microbiology community related to mycobacterial diagnosis, especially for low- and middle-income settings where smear microscopy and culture are still widely used, and where automatic systems like Gene Xpert still are economically challenging.

## INTRODUCTION

Tuberculosis (TB), caused by members of the *Mycobacterium tuberculosis* complex (MTBC), remains one of the leading causes of morbidity and mortality worldwide (1). Despite global efforts to control the disease, TB continues to pose a significant public health burden, especially in low- and middle-income countries (2). The World Health Organization (WHO) reported that approximately 10.8 million individuals worldwide were infected with TB during 2023, marking the highest case count since the WHO began tracking the disease in 1995 (3). In Ecuador, the TB incidence rate reached about 58 cases per 100,000 people, reflecting a concerning 47% rise compared to 2015 (4), the year the End TB Strategy was launched.

On the other hand, non-tuberculous mycobacteria (NTM) comprise mycobacteria that differ from those that cause tuberculosis and leprosy. They are ubiquitous in the environment and mostly harmless to humans. Still, some NTM species can lead to either pulmonary or extrapulmonary infections, including skin and soft tissue, lymphatic, and disseminated infections, as well as nosocomial outbreaks when medical equipment is inadequately disinfected/sterilized (5). NTM infections are rising not only in immunocompromised patients but also in the general population, becoming an emerging public health issue worldwide (6). NTM pulmonary diseases account for most NTM diseases, with the *Mycobacterium avium* (MA) complex, *Mycobacterium abscessus* complex, *Mycobacterium kansasii* (MK), *Mycobacterium malmonense*, and *Mycobacterium xenopi* among the most common pathogens (7).

In this context, early and accurate diagnosis of MTBC and NTM is essential for initiating effective treatment, reducing transmission, and improving patient outcomes. Traditional diagnostic methods, including acid-fast bacilli (AFB) smear microscopy and mycobacterial culture, remain widely used in many settings, particularly in low- and middle-income countries, due to their accessibility and relatively low cost (8–10). However, these methods have notable limitations. Smear microscopy, while rapid, lacks sensitivity (particularly in paucibacillary and HIV-positive patients), while culture, although considered the gold standard, is time consuming and resource intensive (11, 12). Additionally, smear microscopy cannot differentiate among mycobacterial species, while culture may fail to recover fastidious or low-burden species and, on its own, does not provide reliable species-level discrimination without subsequent molecular or biochemical identification (9, 13). In recent years, real-time polymerase chain reaction (PCR) assays have emerged as powerful tools for the rapid detection of TB and NTM. These assays offer improved sensitivity and specificity, shorter turnaround times, and the potential for simultaneous identification and detection of drug resistance (14–17). Several commercial kits are available on the market, yet their performance may vary depending on the population, sample type, and regional mycobacterial diversity (18–20).

In this study, we aimed to evaluate and compare the diagnostic performance of AFB smear microscopy, culture, and three commercial real-time PCR kits: *Viasure MTBC + NTM Real-Time PCR Detection Kit* (CerTest Biotec, Spain), *Mycobacterium Multiplex Nucleic Acid Diagnostic Kit* (Sansure Biotech, Shanghai), and Anyplex MTB/NTMe Real-time Detection (Seegene, South Korea), for the detection of MTBC and NTM in pulmonary, extrapulmonary, and total samples from Ecuadorian patients.

## MATERIALS AND METHODS

### Study design

This study provides a comparative and retrospective evaluation of the clinical performance and diagnostic utility of three Real-Time PCR kits: the *VIASURE M. tuberculosis Complex + Non-Tuberculous Mycobacteria Detection* Kit (hereafter “Viasure”), the *Mycobacterium Multiplex Nucleic Acid Diagnostic* Kit (hereafter “Sansure”), and *Anyplex MTB/NTM Real-time Detection* (hereafter “Seegene”), a widely used molecular test with CE marking. The evaluation was conducted using remnant clinical samples previously analyzed for diagnostic purposes by conventional microbiological methods in various microbiology departments. The primary objective was to assess the accuracy and effectiveness of these PCR kits as diagnostic tools for detecting MTBC and NTM, in comparison with standard diagnostic techniques, within the following schemes:

(1) Scheme 1: Results from the Viasure, Sansure, and Seegene kits were compared with routine diagnostic methods (smear microscopy and culture) for detecting mycobacteria (MTBC or NTM) in pulmonary, extrapulmonary, and total samples.

(2) Scheme 2: Results from smear microscopy, culture, Viasure, Sansure, and Seegene, in pulmonary, extrapulmonary, and total samples, were compared to a composite reference standard (CRS), defined as positive if a sample yielded at least one positive result in any of the evaluated tests; otherwise, it was considered negative. About smear microscopy and culture, the comparison was based on two categories of results: positive for mycobacteria (MTBC or NTM) or negative. With PCR tests, three categories were considered: MTBC, NTM, or negative.

(3) Scheme 3: Results from Viasure and Sansure were compared with those from Seegene in pulmonary, extrapulmonary, and total samples for the detection of MTBC and NTM, excluding positives with Ct ≥ 35. This exclusion criterion was considered as part of an analytical approach aimed at minimizing the impact of low-level amplification near the detection limit. This cutoff was not based on manufacturer-defined reporting criteria, as the assay is designed for qualitative interpretation, but was applied to improve the robustness of comparisons between assays under low-bacterial-load conditions.

The results of the three real-time PCR kits were standardized into three categories: MTBC, NTM, and negative. Clinically, *M. tuberculosis* (MTB) and other members of the complex were considered part of the MTBC category. The smear microscopy and culture techniques had two categories of results: positive (mycobacteria) and negative. Table 1 provides a detailed overview of all comparisons across these three schemes.

**TABLE 1:** Detailed representation of all comparisons made within the four schemes of this study

Scheme	Criteria	Reference test	Origin	Comparison	Target
S1	All samples tested by bacilloscopy and culture.	Bacilloscopy (*n* = 278)	Pulmonary (*n* = 189)	Viasure	Mycobacteria^*d*^
				Sansure	
				Seegene	
			Extrapulmonary (*n* = 89)	Viasure	
				Sansure	
				Seegene	
			Total (*n* = 278)	Viasure	
				Sansure	
				Seegene	
		Culture (*n* = 147)	Pulmonary (*n* = 90)	Viasure	
				Sansure	
				Seegene	
			Extrapulmonary (*n* = 57)	Viasure	
				Sansure	
				Seegene	
			Total (*n* = 147)	Viasure	
				Sansure	
				Seegene	
S2	Composite reference standard (CRS) is considered. This defines a sample as positive if it yielded at least one positive result in any of the evaluated tests.	CRS for samples within the bacilloscopy group (*n* = 278)	Pulmonary (*n* = 189)	Bacilloscopy	Mycobacteria^*d*^
			Extrapulmonary (*n* = 89)	Bacilloscopy	
			Total (*n* = 278)	Bacilloscopy	
		CRS for samples within culture group (*n* = 147)	Pulmonary (*n* = 90)	Culture	
			Extrapulmonary (*n* = 57)	Culture	
			Total (*n* = 147)	Culture	
		CRS (*n* = 407)^*a*^	Pulmonary (*n* = 308)	Viasure	MTBC
				Sansure	
				Seegene	
			Extrapulmonary (*n* = 99)	Viasure	
				Sansure^*c*^	
				Seegene	
			Total (*n* = 407)	Viasure	
				Sansure^*c*^	
				Seegene	
		CRS (*n* = 407)^*b*^	Pulmonary (*n* = 308)	Viasure	
				Sansure	
				Seegene	
			Extrapulmonary (*n* = 99)	Viasure	
				Sansure^*c*^	
				Seegene	
			Total (*n* = 407)	Viasure	
				Sansure^*c*^	
				Seegene	
		CRS (*n* = 407)^*a*^	Pulmonary (*n* = 308)	Viasure	NTM
				Sansure	
				Seegene	
			Extrapulmonary (*n* = 99)	Viasure	
				Sansure^*c*^	
				Seegene	
			Total (*n* = 407)	Viasure	
				Sansure^*c*^	
				Seegene	
		CRS (*n* = 407)^*b*^	Pulmonary (*n* = 308)	Viasure	
				Sansure	
				Seegene	
			Extrapulmonary (*n* = 99)	Viasure	
				Sansure^*c*^	
				Seegene	
			Total (*n* = 407)	Viasure	
				Sansure^*c*^	
				Seegene	
S3	Seegene real-time PCR kit was considered as the reference, considering the exclusion criteria of positives with Ct ≥ 35.	Seegene (*n* = 407)	Pulmonary (*n* = 308)	Viasure	MTBC
				Sansure	
			Extrapulmonary (*n* = 99)	Viasure	
				Sansure^*c*^	
			Total (*n* = 407)	Viasure	
				Sansure^*c*^	
			Pulmonary (*n* = 308)	Viasure	NTM
				Sansure	
			Extrapulmonary (*n* = 99)	Viasure	
				Sansure^*c*^	
			Total (*n* = 407)	Viasure	
				Sansure^*c*^	

^*a*^ In these comparisons targeting MTBC, NTM results were considered negative.

^*b*^ In these comparisons targeting NTM, MTBC results were considered negative.

^*c*^ In this comparison with Sansure, there is one less sample for analysis because one result was invalid.

^*d*^ In these PCR kits comparisons, a result of MTBC or NTM is collectively considered mycobacteria because smear microscopy and culture are the reference tests.

### Sample collection

This study utilized 407 clinical specimens. Of these, 308 were of pulmonary origin, and 99 were non-respiratory specimens collected for the detection of pulmonary and extrapulmonary *M. tuberculosis*, respectively. These results, by origin, are summarized in Table 2. Not all samples included in the study had results available for both smear microscopy and culture. Of the 407 specimens, 278 were tested by smear microscopy and 147 by culture, with an overlap of 18 samples with results from both methods. As a result, smear microscopy and culture were treated as partially overlapping reference tests rather than mutually exclusive data sets in scheme 1.

**TABLE 2:** Clinical specimens in this study

Origin	Specimens	No. (%)
Pulmonary	Sputum	297 (72.97%)
	Pleural fluid	5 (1.23%)
	Induced sputum	2 (0.49%)
	Tracheal aspirates	2 (0.49%)
	Bronchial aspirate	1 (0.25%)
	Bronchial lavage	1 (0.25%)
	Total	308 (75.68%)
Extrapulmonary	Feces	34 (8.35%)
	Urine	22 (5.41%)
	Bone marrow aspirates	15 (3.69%)
	Cerebrospinal fluid samples	9 (2.21%)
	Ascitic fluid	3 (0.74%)
	Gastric aspirates	3 (0.74%)
	Ganglion biopsies	2 (0.49%)
	Inguinal lymph node	3 (0.74%)
	Pericardial fluid	3 (0.74%)
	Unclassified samples	9 (2.21%)
	Total	99 (24.32%)
	Overall total	407 (100%)

At the *Instituto Nacional de Salud Pública e Investigación* (INSPI), samples were collected from adult male and female patients presenting with signs and symptoms of lower respiratory tract infection. Inclusion criteria required a formal request for *M. tuberculosis* identification, preferably through AFB smear microscopy and culture, or, in emergency cases, via GeneXpert assays (personal communication to the authors). All specimens were collected before treatment or drug administration. Following routine diagnostic procedures at the hospital, the remnant specimens were stored adequately at −20°C until further analysis. The samples were collected during a 2-month period. All samples were processed for PCR kits simultaneously, so no more than one thawing cycle and no more than 2 months of −20°C storage were applied to any sample.

### AFB smear microscopy

A direct smear was prepared by spreading a small amount of the clinical specimen onto a clean glass slide and allowing it to air dry. The smear was then heat-fixed by briefly passing the slide over a flame. Staining was performed using the Ziehl–Neelsen method: slides are flooded with carbol fuchsin, heated gently until steaming for 5 min, then rinsed with water. Decolorization is then performed using 3% acid-alcohol for 2 min. Afterward, the slide is rinsed again and counterstained with methylene blue for 1 min. After a final rinse and air-drying, the smear is examined under a light microscope with a 100 × oil-immersion objective. Acid-fast bacilli appear as bright red rods against a blue background (21). AFB smear results obtained from the INSPI as part of routine diagnostic procedures were reported only qualitatively (positive/negative), without gradation of bacillary load (e.g., rare, few, or many), which precluded stratified analysis of PCR performance by smear positivity.

### Culture on Löwenstein-Jensen

Clinical samples were cultured on Löwenstein-Jensen medium, a slow-growing, egg-based medium commonly used for the isolation of *Mycobacterium tuberculosis*. Growth on solid media takes 2–8 weeks due to MTB’s slow replication rate (typically 15–20 h per division). The clinical samples from both groups (pulmonary and extrapulmonary) were decontaminated using the N-acetyl-L-cysteine and sodium hydroxide (NALC-NaOH) method described by Kubica (1963) (22). The processing of sputum samples for MTBC culture using the NALC-NaOH method is widely adopted for decontamination and liquefaction. This method ensures that the sample is free from contaminating bacteria and other microorganisms while maintaining the viability of the mycobacteria. Briefly, fresh samples were collected from the patients in sterile containers. A solution containing N-acetyl-L-cysteine (NALC), a mucolytic agent, and sodium hydroxide (NaOH), a decontaminant, was freshly prepared. NALC liquefies sputum, making it less viscous, while NaOH eliminates contaminating organisms. Equal volumes of the sputum sample and the NALC-NaOH solution (4% NaOH) were mixed in the sterile container. This mixture was incubated at room temperature (22°C–25°C) for 15 min. During that time, NaOH kills non-mycobacterial organisms, and NALC breaks down the mucus, releasing the bacteria into suspension. Following the incubation period, the alkaline environment was neutralized by adding phosphate buffer (pH 6.8) to stop further action of NaOH and prevent harm to MTB. This neutralization step is critical for preserving the viability of the mycobacteria. The entire mixture was then centrifuged at 3,000 × *g* for 15–20 min. High-speed centrifugation concentrates mycobacteria in the pellet, while the supernatant, which contains debris and other contaminants, is discarded. The pellet containing the concentrated mycobacteria was resuspended in a small volume (1–2 mL) of phosphate buffer. This final suspension was used for AFB smear microscopy and inoculated onto culture media.

Extrapulmonary samples were processed following the same general procedures described for respiratory specimens, without additional decontamination or modification steps, as these samples were considered non-viscous and did not require specific pre-treatment.

All specimen handling was performed within a certified biosafety cabinet under BSL-2+ conditions. Centrifugation steps were carried out using sealed rotors, which were only opened within the biosafety cabinet to minimize the risk of aerosol exposure.

### Mycobacteria culture, heat inactivation, and DNA isolation

Mycobacterial colonies were harvested from cultures and resuspended in Tris-EDTA buffer (10 mM Tris–HCl, 1 mM EDTA, pH 8.0). The suspensions were then heat-inactivated at 95°C for 15 min. Following inactivation, samples were centrifuged at 10,000 g for 5 min, and the resulting supernatant was used directly for downstream molecular procedures, as previously described (23–26). All heat-inactivation steps were conducted within the BSL-2+ facility of the *Centro Nacional de Referencia para Micobacterias* at INSPI, following recommended biosafety guidelines to minimize occupational exposure risk (27).

### Viasure kit operation

The *VIASURE M. tuberculosis Complex + Non-Tuberculous Mycobacteria Real-Time PCR Detection* Kit is designed to detect and differentiate DNA from MTBC species, NTM, and *M. tuberculosis*. MTBC is detected by amplifying the IS6110 and IS1081 insertion sequences, which are measured in the FAM channel. The genus *Mycobacterium* is detected in the ROX channel by targeting the 16S rRNA gene, while *M. tuberculosis* is specifically identified via a Cy5-labeled probe targeting the TbD1 region deletion. An internal control, detected in the HEX channel, is included in each reaction to confirm the absence of PCR inhibitors. Both assays use a one-step real-time PCR format. Each reaction well contains all necessary components in a stabilized format, including specific primers and probes, dNTPs, buffer, DNA polymerase, and internal control. This ready-to-use format ensures consistent performance and simplifies workflow for accurate and efficient detection (28).

### Sansure kit operation

The *Mycobacterium Multiplex Nucleic Acid Diagnostic* Kit (Multiplex PCR-Fluorescence Probing) is designed to detect DNA from seven clinically relevant mycobacterial species: MTB, MK, MA, MF (unspecified), *Mycobacterium abscessus* subsp. *abscessus* (MAA), *Mycobacterium abscessus* subsp. *massiliense* (MAM), and *Mycobacterium intracellulare* (MI). The assay combines two molecular detection strategies: (i) real-time fluorescence-based detection using specific probes for certain targets, and (ii) melting curve analysis for the differentiation of others. This dual approach enables the simultaneous identification of multiple targets within a single fluorescence channel.

The Mycobacterium Multiplex Nucleic Acid Diagnostic Kit (Multiplex PCR – Fluorescence Probing) features an open-format design, allowing flexibility in reagent volumes while maintaining the manufacturer-specified final concentrations. Each reaction contained 13.28 μL of MTB/NTM PCR Mix, 0.23 μL of MTB/NTM Enzyme Mix, and 1.5 μL of DNA from the clinical sample. Positive and negative controls were included using 1.5 μL of each DNA preparation. The final reaction volume was 15 μL per tube. PCR amplification was carried out under the following cycling conditions: an initial hold at 50°C for 2 min, followed by 94°C for 3 min; then 45 cycles of 94°C for 10 s, 60°C for 20 s, and 75°C for 20 s, with fluorescence acquisition at the final step of each cycle. This was followed by a melting curve analysis from 62°C to 75°C. Fluorescence detection was channel specific: MF was detected in the ROX channel (Ct ≤ 39), MK in the FAM channel, MA in the HEX channel, and the internal control in the Cy5 channel. Melting curve analysis enabled species differentiation based on melting temperature (Tm): MI in the ROX channel (Tm peak: 70.5°C ± 1°C), MAA in the FAM channel (Tm peak: 69.5°C ± 1°C), MAM in the Cy5 channel (Tm peak: 68°C ± 1°C), and MTB in the HEX channel (Tm peak: 67°C ± 1°C) (29).

### Seegene kit operation

The *Anyplex MTB/NTM Real-time Detection assay* (Seegene) simultaneously detects and differentiates MTB from NTM, utilizing Seegene’s proprietary Dual Priming Oligonucleotide (DPO) technology. The total number of samples was reanalyzed using this assay, in parallel with the Viasure and Sansure kits. For the Seegene assay, PCR amplification was performed according to the manufacturer’s recommended protocol. Each *Anyplex MTB/NTM* reaction included 5 μL of extracted DNA, added to a 15 μL master mix composed of 10 μL of 2 × Anyplex PCR master mix, 3 μL of methoxypsoralen (8-MOP), and 2 μL of 10 × MTB/NTM oligonucleotide mix. Amplification and detection were performed using the CFX96 Real-Time PCR Detection System (Bio-Rad). MTB detection targeted the IS6110 and MPB64 genes, while NTM detection used a specific region of the 16S rRNA gene. Each run included internal extraction controls and the positive and negative amplification controls provided with the kit. An internal control included in the master mix was used to monitor PCR inhibition. Result interpretation was automatically conducted using the system’s software, applying the manufacturer-defined threshold and cutoff values (30).

### Use of molecular assays in non-validated specimen types

Although the manufacturer’s instructions for the Seegene and Viasure assays specify validation for a limited range of specimen types (e.g., sputum, bronchial samples, tissue, and culture isolates), no independent in-house validation was performed for other extrapulmonary sample types included in this study (such as urine, feces, gastric aspirates, and other body fluids). According to information provided by the manufacturers, the listed specimen types correspond to those internally validated and do not preclude the use of additional clinical matrices. Furthermore, all assays incorporate an internal amplification control to monitor potential PCR inhibition, reducing the likelihood of false-negative results due to sample-specific inhibitors.

### Cross-reactivity experiments

To assess the analytical specificity of the three real-time PCR kits considered in this study, 15 samples each of *Haemophilus influenzae*, *Streptococcus pneumoniae*, and *Moraxella catarrhalis* were tested.

### Statistical analysis

Overall agreement, sensitivity, specificity, positive predictive value (PPV), and negative predictive value (NPV) were calculated for comparisons between the different tests and the reference methods, with 95% confidence intervals. These calculations were performed using R software version 2024.4.2.764.1, accessed through the Posit platform.

## RESULTS

As previously mentioned, this study included 407 clinical specimens, comprising 308 respiratory samples (pulmonary) and 99 non-respiratory samples (extrapulmonary), as listed in Table 2. Of the 407 samples, 278 were evaluated by smear microscopy and 147 by culture; additionally, samples within these two groups were tested using the three real-time PCR kits considered.

Of the 278 samples diagnosed by smear microscopy, 41 were positive: 34 pulmonary and 7 extrapulmonary. Within this set, the Viasure kit detected the highest number of positives (167/278), followed by Seegene (148/278) and, finally, Sansure (52/278). This same trend was observed when analyzing only the pulmonary samples: Viasure identified 132/189 positives, Seegene 116/189, and Sansure 46/189. Similarly, in the extrapulmonary samples, Viasure detected 35/89 positives, Seegene 32/89, and Sansure 9/89.

On the other hand, among the 147 culture-diagnosed samples, 48 were positive: 37 pulmonary and 11 extrapulmonary. In this group, Seegene was the kit that detected the most positives (74/147), followed closely by Viasure (72/147) and, finally, Sansure (31/147). A similar pattern was observed in the pulmonary samples: Seegene identified 57/90 positives, Viasure 56/90, and Sansure 25/90. Similarly, in the extrapulmonary samples, Seegene detected 17/57 positives, followed by Viasure (16/57) and Sansure (6/57).

The positivity results, along with the 95% CI and the breakdown by pulmonary, extrapulmonary, and total samples, are detailed in Table 3.

**TABLE 3:** General results of the clinical specimens, along with the calculation of the 95% CI and the breakdown of results by pulmonary, extrapulmonary, and total samples

Group	Total valid samples	Test	Pulmonary sample positives (MTBC + NTM)^*a*^	95% CI for pulmonary samples	Extrapulmonary sample positives (MTBC + NTM)^*a*^	95% CI for extrapulmonary samples	Total positives (pulmonary + extrapulmonary)^*a*^	95% CI for total positives
Smear microscopy diagnosis	278 (pulmonary = 189, extrapulmonary = 89)	Smear microscopy	34/189 (17.99%)	12.7%–23.2%	7/89 (7.87%)	3.86%–15.36%	41/278 (14.75%)	10.58%–18.92%
		Viasure real-time PCR	132/189 (69.84%)	63.2%–76.0%	35/89 (39.33%)	29.82%–49.71%	167/278 (60.07%)	54.31%–65.83%
		Seegene real-time PCR	116/189 (61.38%)	54.4%–68.1%	32/89 (35.96%)	26.76%–46.31%	148/278 (53.24%)	47.37%–59.11%
		Sansure real-time PCR	43/189 (22.75%)	16.9%–28.6%	9/89 (10.11%)	5.41%–18.11%	52/278 (18.71%)	14.12%–23.30%
Culture diagnosis	147 (pulmonary = 90, extrapulmonary = 57)	Culture	37/90 (41.11%)	30.9%–51.9%	11/57 (19.30%)	11.13%–31.34%	48/147 (32.65%)	25.07%–40.23%
		Viasure real-time PCR	56/90 (62.22%)	51.8%–72.0%	16/57 (28.07%)	18.08%–40.83%	72/147 (48.98%)	40.90%–57.06%
		Seegene real-time PCR	57/90 (63.33%)	52.9%–72.9%	17/57 (29.82%)	19.53%–42.66%	74/147 (50.34%)	42.26%–58.42%
		Sansure real-time PCR	25/90 (27.78%)	18.9%–37.9%	6/57 (10.52%)	4.91%–21.12%	31/147 (21.09%)	14.50%–27.68%

^*a*^ In smear microscopy and culture, positive results are related to mycobacteria. In the case of culture, although differences between MTBC and NTM colonies may be observed, additional testing is required for confirmation. Therefore, in the context of this study, we consider positive culture results to be mycobacteria, in general terms.

Using results from routine diagnostic methods (smear microscopy and culture), the CRS, and the Seegene kit, diagnostic performance metrics were calculated for the three schemes detailed in the methodology, separately for pulmonary, extrapulmonary, and total samples (see Table 1). These included overall agreement, sensitivity, specificity, PPV, and NPV, each with corresponding 95% confidence intervals (Tables 4–6).

**TABLE 4:** Confusion matrices for scheme 1, considering smear microscopy as the reference test^*a*^^,^^*b*^

		Smear microscopy								
		Pulmonary			Extrapulmonary			Total samples		
		+	−	Total	+	−	Total	+	−	Total
Viasure	+	31	101	132	7	28	35	38	129	167
	−	3	54	57	0	54	54	3	108	111
	Total	34	155	189	7	82	89	41	237	278
Sansure	+	24	19	43	3	6	9	27	25	52
	−	10	136	146	4	76	80	14	212	226
	Total	34	155	189	7	82	89	41	237	278
Seegene	+	28	88	116	6	26	32	34	114	148
	−	6	67	73	1	56	57	7	123	130
	Total	34	155	189	7	82	89	41	237	278

^*a*^ VIASURE *M. tuberculosis complex + non-tuberculous mycobacteria* Real-Time PCR Detection Kit (Viasure), *Mycobacterium Multiplex Nucleic Acid Diagnostic* Kit results (Sansure), and *Anyplex MTB/NTMe Real-time Detection* (Seegene), compared to routine diagnosis by smear microscopy in pulmonary, extrapulmonary, and total samples for mycobacteria detection.

^*b*^ +, positive; −, negative.

**TABLE 5:** Confusion matrices for scheme 1, considering culture as the reference test^*a*^^,^^*b*^

		Culture								
		Pulmonary			Extrapulmonary			Total samples		
		+	−	Total	+	−	Total	+	−	Total
Viasure	+	30	26	56	9	7	16	39	33	72
	–	7	27	34	2	39	41	9	66	75
	Total	37	53	90	11	46	57	48	99	147
Sansure	+	20	5	25	3	3	6	23	8	31
	–	17	48	65	8	43	51	25	91	116
	Total	37	53	90	11	46	57	48	99	147
Seegene	+	30	27	57	7	10	17	37	37	74
	–	7	26	33	4	36	40	11	62	73
	Total	37	53	90	11	46	57	48	99	147

^*a*^ VIASURE *M. tuberculosis complex + non-tuberculous mycobacteria* Real-Time PCR Detection Kit (Viasure), *Mycobacterium Multiplex Nucleic Acid *Diagnostic Kit results (Sansure), and *Anyplex MTB/NTMe *Real-Time Detection (Seegene), compared to routine diagnosis by culture in pulmonary, extrapulmonary, and total samples for mycobacteria detection.

^*b*^ +, positive; −, negative.

**TABLE 6:** Diagnostic performance measures of scheme 1^*a*^

PCR kit	Reference test	Origin	TP	TN	FP	FN	Overall agreement	Sensitivity	Specificity	PPV	NPV
Viasure	Smear microscopy	Pulmonary	31	54	101	3	0.45 (0.38–0.521)	0.912 (0.77–0.97)	0.348 (0.278–0.426)	0.235 (0.171–0.314)	0.947 (0.856–0.982)
Sansure	Smear microscopy	Pulmonary	24	136	19	10	0.847 (0.788–0.891)	0.706 (0.538–0.832)	0.877 (0.817–0.92)	0.558 (0.411–0.696)	0.932 (0.878–0.962)
Seegene	Smear microscopy	Pulmonary	28	67	88	6	0.503 (0.432–0.573)	0.824 (0.665–0.916)	0.432 (0.357–0.511)	0.241 (0.173–0.327)	0.918 (0.832–0.962)
Viasure	Smear microscopy	Extrapulmonary	7	54	28	0	0.685 (0.583–0.772)	1 (0.646–1)	0.659 (0.551–0.752)	0.2 (0.1–0.359)	1 (0.934–1)
Sansure	Smear microscopy	Extrapulmonary	3	76	6	4	0.888 (0.805–0.938)	0.429 (0.158–0.75)	0.927 (0.849–0.966)	0.333 (0.121–0.646)	0.95 (0.878–0.98)
Seegene	Smear microscopy	Extrapulmonary	6	56	26	1	0.697 (0.595–0.782)	0.857 (0.487–0.974)	0.683 (0.576–0.773)	0.188 (0.089–0.353)	0.982 (0.907–0.997)
Viasure	Smear microscopy	Total	38	108	129	3	0.525 (0.466–0.583)	0.927 (0.806–0.975)	0.456 (0.394–0.519)	0.228 (0.17–0.297)	0.973 (0.923–0.991)
Sansure	Smear microscopy	Total	27	212	25	14	0.86 (0.814–0.896)	0.659 (0.505–0.784)	0.895 (0.849–0.927)	0.519 (0.387–0.649)	0.938 (0.899–0.963)
Seegene	Smear microscopy	Total	34	123	114	7	0.565 (0.506–0.622)	0.829 (0.687–0.915)	0.519 (0.456–0.582)	0.23 (0.169–0.304)	0.946 (0.893–0.974)
Viasure	Culture	Pulmonary	30	27	26	7	0.633 (0.53–0.726)	0.811 (0.658–0.905)	0.509 (0.379–0.639)	0.536 (0.407–0.66)	0.794 (0.632–0.896)
Sansure	Culture	Pulmonary	20	48	5	17	0.756 (0.657–0.833)	0.541 (0.384–0.69)	0.906 (0.797–0.959)	0.8 (0.609–0.911)	0.738 (0.621–0.83)
Seegene	Culture	Pulmonary	30	26	27	7	0.622 (0.519–0.715)	0.811 (0.658–0.905)	0.491 (0.361–0.621)	0.526 (0.399–0.65)	0.788 (0.623–0.893)
Viasure	Culture	Extrapulmonary	9	39	7	2	0.842 (0.726–0.915)	0.818 (0.523–0.949)	0.848 (0.718–0.924)	0.562 (0.332–0.769)	0.951 (0.839–0.987)
Sansure	Culture	Extrapulmonary	3	43	3	8	0.807 (0.687–0.889)	0.273 (0.098–0.566)	0.935 (0.825–0.978)	0.5 (0.188–0.812)	0.843 (0.72–0.918)
Seegene	Culture	Extrapulmonary	7	36	10	4	0.754 (0.629–0.848)	0.636 (0.354–0.848)	0.783 (0.644–0.877)	0.412 (0.216–0.64)	0.9 (0.769–0.96)
Viasure	Culture	Total	39	66	33	9	0.714 (0.636–0.781)	0.812 (0.681–0.898)	0.667 (0.569–0.752)	0.542 (0.427–0.652)	0.88 (0.787–0.936)
Sansure	Culture	Total	23	91	8	25	0.776 (0.702–0.835)	0.479 (0.345–0.617)	0.919 (0.849–0.959)	0.742 (0.568–0.863)	0.784 (0.701–0.85)
Seegene	Culture	Total	37	62	37	11	0.673 (0.594–0.744)	0.771 (0.635–0.867)	0.626 (0.528–0.715)	0.5 (0.389–0.611)	0.849 (0.75–0.914)

^*a*^ Overall agreement, true positive (TP), true negative (TN), false positive (FP), false negative (FN), sensitivity, specificity, positive predictive value (PPV), and negative predicted value (NPV) of VIASURE *M. tuberculosis complex + non-tuberculous mycobacteria* Real-Time PCR Detection Kit (Viasure), *Mycobacterium Multiplex Nucleic Acid Diagnostic *Kit (Sansure), and *Anyplex MTB/NTMe *Real-time Detection (Seegene) compared to routine diagnosis by culture and smear microscopy in pulmonary, extrapulmonary, and total samples for mycobacteria detection. The 95% confidence intervals are described in parentheses.

### Scheme 1: PCR real-time kits comparison with smear microscopy and culture

Of the 407 samples evaluated, 278 were examined by smear microscopy, and 147 were routinely tested by culture. Among these, 41 were smear microscopy positive, 237 were smear microscopy negative, 48 samples were culture positive, and 99 were culture negative. Notably, 15 samples tested positive for culture but were negative for smear microscopy. The performance of the Viasure, Sansure, and Seegene kits was assessed in comparison to smear microscopy and culture (Tables 4–6).

### Viasure kit results

#### Samples with smear microscopy diagnosis

Of the 278 samples analyzed by smear microscopy during routine diagnosis, 189 were pulmonary, and 89 were extrapulmonary. Among the pulmonary samples, the Viasure kit identified 120 MTBC- and 12 NTM-positive samples, totaling 132 mycobacterial isolates. Among the extrapulmonary samples, the Viasure kit identified 27 MTBC-positive samples and 3 NTM-positive samples, for a total of 30 mycobacterial samples (see Tables 4 and 6).

#### Samples with culture diagnosis

Of the 147 samples analyzed by culture during routine diagnosis, 90 were from the pulmonary system, and 57 were from extrapulmonary sites. Among the pulmonary samples, the Viasure kit identified 47 as positive for MTBC and 9 as positive for NTM, for a total of 56 mycobacteria. Among the extrapulmonary samples, the Viasure kit identified 14 MTBC-positive samples and 2 NTM-positive samples; therefore, 16 samples were mycobacteria (see Tables 5 and 6).

### Sansure kit results

#### Samples with smear microscopy diagnosis

Of the 278 samples analyzed by smear microscopy during routine diagnosis, 189 were pulmonary, and 89 were extrapulmonary. Among the pulmonary samples, the Sansure kit identified 35 MTBC-positive and 8 NTM-positive samples, for a total of 43 mycobacterial isolates. Among the extrapulmonary samples, this kit identified seven MTBC-positive samples and two NTM-positive samples; therefore, nine were mycobacteria (see Tables 4 and 6).

#### Samples with culture diagnosis

Of the 147 samples analyzed by culture in routine diagnosis, 90 were pulmonary, and 57 were extrapulmonary. These groups were analyzed separately. In diagnostics with this kit, one sample returned an invalid result because none of the markers amplified. Among the pulmonary samples, the Sansure kit identified 22 MTBC-positive and 3 NTM-positive samples, for a total of 25 mycobacterial isolates. Among the extrapulmonary samples, this kit identified five MTBC-positive and two NTM-positive samples; therefore, seven samples were mycobacterial (see Tables 5 and 6).

### Seegene kit results

#### Samples with smear microscopy diagnosis

Of the 278 samples analyzed by smear microscopy during routine diagnosis, 189 were pulmonary, and 89 were extrapulmonary. Among the pulmonary samples, the Seegene kit identified 114 MTBC-positive and 2 NTM-positive samples, for a total of 116 mycobacterial isolates. Among the extrapulmonary samples, this kit identified 32 MTBC-positive samples and none NTM positive; therefore, 32 were mycobacteria (see Tables 4 and 6).

#### Samples with culture diagnosis

As mentioned earlier, of the 147 samples analyzed by culture in routine diagnosis, 90 were pulmonary, and 57 were extrapulmonary. Among the pulmonary samples, the Seegene kit identified 55 MTBC-positive and 2 NTM-positive samples, for a total of 57 mycobacteria. Among the extrapulmonary samples, the Seegene kit identified 17 MTBC-positive samples and no NTM-positive; therefore, 17 mycobacteria are considered here (see Tables 5 and 6).

### Scheme 2: PCR real-time kits, culture, smear microscopy compared to the CRS

For the CRS, we assigned a positive result to a strain (MTN or NTM) if at least one test was positive; otherwise, we assigned a negative result (Tables 7–9). Thus, among the 407 samples in the study considered within this scheme, 303 were positive for MTBC, 16 for NTM, and 88 were negative. Under this criterion, the two routine diagnostic tests (smear microscopy and culture) and the three real-time PCR kits were compared against the CRS. For smear microscopy and culture comparisons, an MTBC or NTM result was considered mycobacterial (see Tables 7 and 10).

**TABLE 7:** Confusion matrices of routine diagnostic tests for scheme 2, considering CRS as the reference test^*a*^^,^^*b*^

		CRS								
		Pulmonary			Extrapulmonary			Total samples		
		+	−	Total	+	−	Total	+	−	Total
Smear microscopy	+	34	0	34	7	0	7	41	0	41
	−	125	30	155	45	37	82	170	67	237
	Total	159	30	189	52	37	89	211	67	278
Culture	+	37	0	37	11	0	11	48	0	48
	−	37	16	53	19	27	46	56	43	99
	Total	74	16	90	30	27	57	104	43	147

^*a*^ Smear microscopy and culture compared to CRS in pulmonary, extrapulmonary, and total samples for mycobacteria detection.

^*b*^ +, positive; −, negative.

**TABLE 8:** Confusion matrices of PCR real-time kits for scheme 2, considering CRS as the reference test (MTBC)^*a*^^,^^*b*^

		CRS for MTBC								
		Pulmonary			Extrapulmonary			Total samples		
		+	–	Total	+	–	Total	+	–	Total
Viasure	+	184	0	184	38	0	38	222	0	222
	−	61	63	124	20	41	61	81	104	185
	Total	245	63	308	58	41	99	303	104	407
Sansure	+	56	0	56	7	0	7	63	0	63
	−	189	63	252	50	41	91	239	104	343
	Total	245	63	308	57	41	98	302	104	406
Seegene	+	179	0	179	36	0	36	215	0	215
	−	66	63	129	22	41	63	88	104	192
	Total	245	63	308	58	41	99	303	104	407

^*a*^ VIASURE *M. tuberculosis complex + non-tuberculous mycobacteria Real-Time PCR Detection* Kit (Viasure), *Mycobacterium Multiplex Nucleic Acid Diagnostic* Kit results (Sansure), and *Anyplex MTB/NTMe Real-time Detection* (Seegene), compared to CRS in pulmonary, extrapulmonary, and total samples for mycobacteria detection.

^*b*^ +, positive; −, negative.

**TABLE 9:** Confusion matrices of PCR real-time kits for scheme 2, considering CRS as the reference test (NTM)^*a*^^,^^*b*^

		CRS for NTM								
		Pulmonary			Extrapulmonary			Total samples		
		+	–	Total	+	–	Total	+	–	Total
Viasure	+	12	6	18	2	2	4	14	8	22
	−	1	289	290	1	94	95	2	383	385
	Total	13	295	308	3	96	99	16	391	407
Sansure	+	3	6	9	0	2	2	3	8	11
	−	10	289	299	3	93	96	13	382	395
	Total	13	295	308	3	95	98	16	390	406
Seegene	+	1	1	2	1	0	1	2	1	3
	−	12	294	306	2	96	98	14	390	404
	Total	13	295	308	3	96	99	16	391	407

^*a*^ VIASURE *M. tuberculosis complex + non-tuberculous mycobacteria Real-Time PCR Detection* Kit (Viasure), *Mycobacterium Multiplex Nucleic Acid Diagnostic* Kit results (Sansure), and *Anyplex MTB/NTMe Real-time Detection* (Seegene), compared to CRS in pulmonary, extrapulmonary, and total samples for NTM detection.

^*b*^ +, positive; −, negative.

**TABLE 10:** Diagnostic performance measures of scheme 2—composite reference standard (CRS)^*a*^

Test	Reference test	Origin	Target	TP	TN	FP	FN	Overall agreement	Sensitivity	Specificity	PPV	NPV
Smear microscopy	CRS	Pulmonary	Mycobacteria	34	30	0	125	0.339 (0.275–0.409)	0.214 (0.157–0.284)	1 (0.886–1)	1 (0.898–1)	0.194 (0.139–0.263)
Smear microscopy	CRS	Extrapulmonary	Mycobacteria	7	37	0	45	0.494 (0.393–0.596)	0.135 (0.067–0.253)	1 (0.906–1)	1 (0.646–1)	0.451 (0.348–0.559)
Smear microscopy	CRS	Total	Mycobacteria	41	67	0	170	0.388 (0.333–0.447)	0.194 (0.147–0.253)	1 (0.946–1)	1 (0.914–1)	0.283 (0.229–0.343)
Culture	CRS	Pulmonary	Mycobacteria	37	16	0	37	0.589 (0.486–0.685)	0.5 (0.389–0.611)	1 (0.806–1)	1 (0.906–1)	0.302 (0.195–0.435)
Culture	CRS	Extrapulmonary	Mycobacteria	11	27	0	19	0.667 (0.537–0.775)	0.367 (0.219–0.545)	1 (0.875–1)	1 (0.741–1)	0.587 (0.443–0.717)
Culture	CRS	Total	Mycobacteria	48	43	0	56	0.619 (0.538–0.694)	0.462 (0.369–0.557)	1 (0.918–1)	1 (0.926–1)	0.434 (0.341–0.533)
Viasure	CRS	Pulmonary	MTBC	184	63	0	61	0.802 (0.754–0.843)	0.751 (0.693–0.801)	1 (0.943–1)	1 (0.98–1)	0.508 (0.421–0.595)
Sansure	CRS	Pulmonary	MTBC	56	63	0	189	0.386 (0.334–0.442)	0.229 (0.18–0.285)	1 (0.943–1)	1 (0.936–1)	0.25 (0.201–0.307)
Seegene	CRS	Pulmonary	MTBC	179	63	0	66	0.786 (0.737–0.828)	0.731 (0.672–0.782)	1 (0.943–1)	1 (0.979–1)	0.488 (0.404–0.574)
Viasure	CRS	Extrapulmonary	MTBC	38	41	0	20	0.798 (0.709–0.865)	0.655 (0.527–0.764)	1 (0.914–1)	1 (0.908–1)	0.672 (0.547–0.777)
Sansure	CRS	Extrapulmonary	MTBC	7	41	0	50	0.386 (0.334–0.442)	0.229 (0.18–0.285)	1 (0.943–1)	1 (0.936–1)	0.25 (0.201–0.307)
Seegene	CRS	Extrapulmonary	MTBC	36	41	0	22	0.778 (0.686–0.848)	0.621 (0.492–0.734)	1 (0.914–1)	1 (0.904–1)	0.651 (0.527–0.757)
Viasure	CRS	Total	MTBC	222	104	0	81	0.801 (0.759–0.837)	0.733 (0.68–0.779)	1 (0.964–1)	1 (0.983–1)	0.562 (0.49–0.632)
Sansure	CRS	Total	MTBC	63	104	0	239	0.411 (0.364–0.46)	0.209 (0.167–0.258)	1 (0.964–1)	1 (0.943–1)	0.303 (0.257–0.354)
Seegene	CRS	Total	MTBC	215	104	0	88	0.784 (0.741–0.821)	0.71 (0.656–0.758)	1 (0.964–1)	1 (0.982–1)	0.542 (0.471–0.611)
Viasure	CRS	Pulmonary	NTM	12	289	6	1	0.977 (0.954–0.989)	0.923 (0.667–0.986)	0.98 (0.956–0.991)	0.667 (0.438–0.837)	0.997 (0.981–0.999)
Sansure	CRS	Pulmonary	NTM	3	289	6	10	0.948 (0.917–0.968)	0.231 (0.082–0.503)	0.98 (0.956–0.991)	0.333 (0.121–0.646)	0.967 (0.94–0.982)
Seegene	CRS	Pulmonary	NTM	1	294	1	12	0.958 (0.929–0.975)	0.077 (0.014–0.333)	0.997 (0.981–0.999)	0.5 (0.094–0.905)	0.961 (0.933–0.977)
Viasure	CRS	Extrapulmonary	NTM	2	94	2	1	0.97 (0.915–0.99)	0.667 (0.208–0.939)	0.979 (0.927–0.994)	0.5 (0.15–0.85)	0.989 (0.943–0.998)
Sansure	CRS	Extrapulmonary	NTM	0	93	2	3	0.948 (0.917–0.968)	0.231 (0.082–0.503)	0.98 (0.956–0.991)	0.333 (0.121–0.646)	0.967 (0.94–0.982)
Seegene	CRS	Extrapulmonary	NTM	1	96	0	2	0.98 (0.929–0.994)	0.333 (0.062–0.792)	1 (0.962–1)	1 (0.206–1)	0.98 (0.929–0.994)
Viasure	CRS	Total	NTM	14	383	8	2	0.975 (0.955–0.987)	0.875 (0.64–0.965)	0.98 (0.96–0.99)	0.636 (0.43–0.803)	0.995 (0.981–0.999)
Sansure	CRS	Total	NTM	3	382	8	13	0.948 (0.922–0.966)	0.188 (0.066–0.43)	0.979 (0.96–0.99)	0.273 (0.098–0.566)	0.967 (0.945–0.981)
Seegene	CRS	Total	NTM	2	390	1	14	0.963 (0.94–0.978)	0.125 (0.035–0.36)	0.997 (0.986–1)	0.667 (0.208–0.939)	0.965 (0.943–0.979)

^*a*^ Overall agreement, true positive (TP), true negative (TN), false positive (FP), and false negative (FN), sensitivity, specificity, positive predictive value (PPV), and negative predictive value (NPV) of VIASURE *M. tuberculosis complex + non-tuberculous mycobacteria* Real-Time PCR Detection Kit (Viasure) and *Mycobacterium Multiplex Nucleic Acid Diagnostic *Kit (Sansure) compared to *Anyplex MTB/NTMe *Real-time Detection (Seegene) in pulmonary, extrapulmonary, and total samples. The 95% confidence intervals are described in parentheses.

Of the 308 pulmonary samples, the Viasure kit identified 184 as MTBC and 18 as NTM. In the 99 extrapulmonary samples, Viasure identified 38 as MTBC and 4 as NTM. Concerning the Sansure kit, in the pulmonary group, it identified 56 MTBC and 9 NTM. In the extrapulmonary group, Sansure recognized 7 MTBC and 2 NTM. On the other hand, the Seegene kit identified 179 MTBC and 2 NTM in the pulmonary group. In the extrapulmonary group, it identified 36 MTBC cases and 1 NTM case (see Tables 8–10).

### Scheme 3: Viasure and Sansure comparison to Seegene

Of the 407 samples considered in this study, Seegene identified 213 as positive for MTBC and 3 as positive for NTM; after excluding samples with Ct ≥ 35, we were left with 194 samples: 141 pulmonary and 53 extrapulmonary. Of these, in pulmonary samples, Seegene identified 61 MTBC, 0 NTM, and 80 negatives, and in extrapulmonary samples, 7 MTBC, 0 NTM, and 46 negatives (Tables 11–13).

**TABLE 11:** Confusion matrices for scheme 3, considering Seegene as the reference test for MTBC results^*a*^^,^^*b*^

		Seegene for MTBC								
		Pulmonary			Extrapulmonary			Total samples		
		+	−	Total	+	−	Total	+	−	Total
Viasure	+	59	10	69	7	4	11	66	14	80
	−	2	70	72	0	42	42	2	112	114
	Total	61	80	141	7	46	53	68	126	194
Sansure	+	52	1	53	5	0	5	57	1	58
	−	9	79	88	2	45	47	11	124	135
	Total	61	80	141	7	45	52	68	125	193

^*a*^ VIASURE *M. tuberculosis complex + non-tuberculous mycobacteria* real-time PCR detection kit (Viasure) and *Mycobacterium multiplex nucleic Acid diagnostic* kit results (Sansure) compared to *Anyplex MTB/NTMe Real-time Detection* (Seegene), in pulmonary, extrapulmonary, and total samples for MTBC detection. Here, an NTM-positive result was considered negative for MTBC detection.

^*b*^ +, positive; −, negative.

**TABLE 12:** Confusion matrices for scheme 3, considering Seegene as the reference test for NTM results^*a*^^,^^*b*^

		Seegene for NTM								
		Pulmonary			Extrapulmonary			Total samples		
		+	–	Total	+	–	Total	+	–	Total
Viasure	+	0	8	8	0	1	1	0	9	9
	–	0	133	133	0	52	52	0	185	185
	Total	0	141	141	0	53	53	0	194	194
Sansure	+	0	3	3	0	1	1	0	4	4
	–	0	138	138	0	51	51	0	189	189
	Total	0	141	141	0	52	52	0	193	193

^*a*^ VIASURE *M. tuberculosis complex + non-tuberculous mycobacteria* real-time PCR detection kit (Viasure) and *Mycobacterium multiplex nucleic Acid diagnostic* kit results (Sansure) compared to *Anyplex MTB/NTMe Real-time Detection* (Seegene), in pulmonary, extrapulmonary, and total samples for NTM detection. Here, an MTBC-positive result was considered negative for NTM detection.

^*b*^ +, positive; −, negative.

**TABLE 13:** Diagnostic performance measures of scheme 3^*a*^

Comparison	Reference test	Origin	Target	TP	TN	FP	FN	Overall agreement	Sensitivity	Specificity	PPV	NPV
Viasure	Seegene	Pulmonary	MTBC	59	70	10	2	0.915 (0.857–0.951)	0.967 (0.888–0.991)	0.875 (0.785–0.931)	0.855 (0.753–0.919)	0.972 (0.904–0.992)
Sansure	Seegene	Pulmonary	MTBC	52	79	1	9	0.929 (0.874–0.961)	0.852 (0.743–0.920)	0.988 (0.932–0.998)	0.981 (0.901–0.997)	0.898 (0.817–0.945)
Viasure	Seegene	Extrapulmonary	MTBC	7	42	4	0	0.925 (0.821–0.970)	1.000 (0.646–1.000)	0.913 (0.797–0.966)	0.636 (0.354–0.848)	1.000 (0.916–1.000)
Sansure	Seegene	Extrapulmonary	MTBC	5	45	0	2	0.962 (0.870–0.989)	0.714 (0.359–0.918)	1.000 (0.921–1.000)	1.000 (0.566–1.000)	0.957 (0.858–0.988)
Viasure	Seegene	Total	MTBC	66	112	14	2	0.918 (0.870–0.949)	0.971 (0.899–0.992)	0.889 (0.822–0.933)	0.825 (0.727–0.893)	0.982 (0.938–0.995)
Sansure	Seegene	Total	MTBC	57	124	1	11	0.938 (0.894–0.964)	0.838 (0.733–0.907)	0.992 (0.956–0.999)	0.983 (0.909–0.997)	0.919 (0.860–0.954)
Viasure	Seegene	Pulmonary	NTM	0	133	8	0	0.943 (0.892–0.971)	NaN (NaN–NaN)^*b*^	0.943 (0.892–0.971)	0.000 (0.000–0.324)	1.000 (0.972–1.000)
Sansure	Seegene	Pulmonary	NTM	0	138	3	0	0.979 (0.939–0.993)	NaN (NaN–NaN)	0.979 (0.939–0.993)	0.000 (0.000–0.561)	1.000 (0.973–1.000)
Viasure	Seegene	Extrapulmonary	NTM	0	52	1	0	0.981 (0.901–0.997)	NaN (NaN–NaN)	0.981 (0.901–0.997)	0.000 (0.000–0.793)	1.000 (0.931–1.000)
Sansure	Seegene	Extrapulmonary	NTM	0	51	1	0	0.981 (0.899–0.997)	NaN (NaN–NaN)	0.981 (0.899–0.997)	0.000 (0.000–0.793)	1.000 (0.930–1.000)
Viasure	Seegene	Total	NTM	0	185	9	0	0.954 (0.914–0.975)	NaN (NaN–NaN)	0.954 (0.914–0.975)	0.000 (0.000–0.299)	1.000 (0.980–1.000)
Sansure	Seegene	Total	NTM	0	189	4	0	0.979 (0.948–0.992)	NaN (NaN–NaN)	0.979 (0.948–0.992)	0.000 (0.000–0.490)	1.000 (0.980–1.000)

^*a*^ Overall agreement, true positive (TP), true negative (TN), false positive (FP), false negative (FN), sensitivity, specificity, positive predictive value (PPV), and negative predictive value (NPV) of *VIASURE M. tuberculosis complex + non-tuberculous mycobacteria* real-time PCR detection kit (Viasure) and *Mycobacterium multiplex nucleic Acid Diagnostic Kit* (Sansure) compared to *Anyplex MTB/NTMe Real-time Detection* (Seegene) in pulmonary, extrapulmonary, and total samples. The 95% confidence intervals are described in parentheses.

^*b*^ NaN: metric that could not be calculated.

#### Viasure kit analysis results

After applying the exclusion criteria for positive samples with Ct ≥ 35, 194 samples remained: 80 MTBC, 9 NTM, and 105 negative according to the Viasure kit. Of this group, 141 were pulmonary samples: 69 MTBC, 8 NTM, and 64 negative. The remaining 53 samples were extrapulmonary: 11 were MTBC, 1 was NTM, and 41 were negative (Tables 11–13).

#### Sansure kit analysis results

In this kit, one sample was invalid because none of the markers were amplified. Therefore, a total of 193 samples remained after applying the exclusion criteria. Of these, 58 were identified as MTBC, 4 as NTM, and 131 as negative. Within the pulmonary group (141), 53 were MTBC, 3 were NTM, and 85 were negative. Meanwhile, in the extrapulmonary group (52), 5 were MTBC, 1 was NTM, and 46 were negative (Tables 11–13).

### Cross-reactivity assays

To evaluate the analytical specificity of the three real-time PCR kits, cross-reactivity assays were performed using DNA from 15 strains each of *Haemophilus influenzae*, *Streptococcus pneumoniae*, and *Moraxella catarrhalis*, species commonly associated with respiratory infections and potential sources of nonspecific amplification. All reactions yielded negative results across the three evaluated kits (Viasure, Sansure, and Seegene), indicating the absence of cross-reactivity and confirming the high analytical specificity of the assays for *Mycobacterium tuberculosis complex* and nontuberculous mycobacteria targets.

## DISCUSSION

Mycobacterial infections remain a significant global public health concern, influenced by multiple factors related to the pathogen, host, and environment. These include the intrinsic virulence and antibiotic resistance profiles of mycobacteria, host-related factors such as comorbidities and immune status, and environmental conditions such as poor living standards, socioeconomic inequality, and population displacement or migration (25, 31, 32). Diagnostic efforts are further challenged by the infrastructure, technical expertise, and time required for the microbiological processes involved in sampling, culturing, and identifying mycobacteria (33–35). Accurate identification is essential for selecting the appropriate treatment, as therapeutic regimens differ not only between TB and NTM infections but also among the various NTM species.

In this retrospective-comparative study, the clinical performance of VIASURE *M. tuberculosis complex + non-tuberculous mycobacteria* Real-Time PCR Detection Kit (“Viasure”), *Mycobacterium Multiplex Nucleic Acid Diagnostic Kit* (“Sansure”), *Anyplex MTB/NTMe Real-time Detection* (“Seegene”), smear microscopy, and culture in pulmonary, extrapulmonary, and total samples were evaluated for mycobacteria (MTBC or NTM), MTBC, and NTM diagnosis, under three schemes: (i) PCR real-time kits comparison with smear microscopy and culture; (ii) PCR real-time kits, smear microscopy, and culture compared to a composite reference standard; and (iii) Viasure and Sansure comparison to the Seegene kit, excluding positive results with a Ct ≥ 35.

Analysis of the overall positivity results (Table 3) shows that the hierarchy between methods is very clear. Smear microscopy has the lowest yield (around 15% overall), culture detects about a third of cases, and the real-time PCR assays sit well above both. Among the PCRs (see Table 3), Viasure has the highest overall positivity considering total samples (60.07% in the smear microscopy diagnosis group and 48.98% in the culture diagnosis group), followed by Seegene (53.24% in the smear microscopy diagnosis group and 50.34% in the culture diagnosis group), while Sansure is only slightly above smear (18.71% vs 14.75%) and even below culture (21.09% vs 32.65%). The same pattern appears in pulmonary and extrapulmonary subsets: Viasure and Seegene pick up two to three times more positives than culture and several times more than smear, whereas Sansure behaves almost like a non-molecular method, missing many cases that the other PCRs detect.

Scheme 1, which uses smear and culture as references, converts those proportions into diagnostic profiles. Against smear, Viasure and Seegene show very high sensitivity but low specificity because they were positive for many smear-negative samples. Sansure shows the opposite behavior: high specificity and agreement, but clearly lower sensitivity, essentially mirroring smear rather than adding much beyond it. When culture is the reference, Viasure and Seegene again detect most culture-positive pulmonary and extrapulmonary cases but disagree more often on culture-negative samples, whereas Sansure aligns closely with culture but misses a substantial fraction of culture-confirmed TB. In summary, the results from Scheme 1 align with the data shown in Table 3, and the reduced specificity of Viasure and Seegene is an artifact of the lower sensitivity of smear and culture in detecting true-positive samples.

Scheme 2, using a composite reference standard, makes the contrast even sharper. Once a case is considered positive, if *any* test is positive, smear and culture are obviously very insensitive: smear recovers only about 15%–20% of CRS-positive infections, whereas culture recovers about 40%–50%. In that framework, Viasure and Seegene emerge as the real “workhorses” for MTBC, with good sensitivities (around 70%–75%), accounting for most of the mycobacterial burden in the cohort. Sansure remains markedly under-sensitive for MTBC. For NTM, Viasure appears to detect a higher proportion of CRS-positive NTM cases, while Seegene and Sansure remain highly specific but miss a substantial fraction of NTM infections recognized by the CRS. However, these findings should be interpreted with caution, as NTM identification at the culture level was not performed in this study, and culture-positive NTM cases were inferred based on concordance with molecular results. Therefore, species-specific performance and true specificity for individual NTM organisms could not be assessed.

Scheme 3 focuses on inter-kit agreement, using Seegene as a practical reference, and excludes positives with Ct ≥ 35. In this Ct range, concordance for MTBC is excellent: both Viasure and Sansure agree with Seegene in the great majority of cases. Viasure is almost completely sensitive to Seegene’s MTBC positives but calls for a few additional positives; Sansure almost never disagrees with Seegene negatives but misses more of its positives. This supports the idea that excluding high-Ct results tightens concordance, particularly for MTBC. For NTM, however, the same filter removes all Seegene NTM positives, so the apparent loss of sensitivity in Scheme 3 simply reflects the way the reference was defined and is not informative about real NTM performance. Regarding this scheme, the use of the Seegene assay as a reference comparator was primarily based on its widespread use, CE-marking, and the substantial body of published evidence supporting its performance in mycobacterial detection (36–42). The exclusion of results with Ct values ≥ 35 should be interpreted as an analytical decision to enhance comparability between methods, rather than as a reflection of the assay’s intended qualitative reporting framework. On the other hand, although the manufacturer’s instructions specify validation for a limited range of specimen types, its inclusion in this study was intended to provide a practical benchmark for comparison across assays in a real-world diagnostic setting.

Overall, the three pieces of analysis converge on a consistent message: Viasure and Seegene serve as high-yield molecular tests for TB, with Viasure showing the highest apparent yield for NTM detection, while Sansure is a very specific but much less sensitive option that behaves more like traditional methods than it does in overcoming their limitations. It is important to note that although the Sansure assay can differentiate several NTM species, its performance on direct clinical samples in this study was limited, precluding meaningful species-level identification. This contrasts with previous work using culture isolates, in which Sansure demonstrated adequate performance for NTM species identification, suggesting that its utility may depend strongly on sample type and bacterial load (36).

Across different settings, the Seegene Anyplex MTB/NTM assay is generally described as a high-specificity test with good sensitivity for MTBC and more variable performance for NTM, particularly in paucibacillary or extrapulmonary disease (37–40). Reported MTBC sensitivities typically fall in the mid-80% range with specificities ≥95% in respiratory samples, while NTM sensitivities are often clearly lower but with very high specificity (39–41). More extreme paucibacillary scenarios, such as tuberculous meningitis, show that Anyplex can maintain 100% specificity but with clinical sensitivities as low as ~30%–50%, highlighting its value as a “rule-in” rather than “rule-out” tool in those contexts (42). Against this backdrop, our MTBC results for Seegene in schemes 1 and 2 fit within the expected “high specificity, mid-to-high sensitivity” window, but under more demanding conditions (all clinical samples, including many extrapulmonary and low-bacillary specimens). This prior evidence that Seegene is a robust benchmark motivated the design of scheme 3, where it was used as a reference (excluding positive samples with Ct ≥ 35), confirming high inter-kit concordance for MTBC once very high-Ct signals were excluded.

At the same time, our data add a distinct perspective on NTM and on the comparative performance of Viasure and Sansure. While several studies show that Seegene and other commercial assays can detect a broad panel of NTMs, they also report relatively modest sensitivity to NTMs in direct clinical samples (39–41). In our cohort, this limitation was even more pronounced: under the CRS, Seegene showed high specificity but low sensitivity for NTM, and after applying the Ct ≥ 35 exclusion in scheme 3, virtually all Seegene NTM signals disappeared, making NTM metrics vs Seegene difficult to interpret. In contrast, Viasure consistently combined high sensitivity and high specificity for both MTBC and NTM, whereas Sansure behaved as a very specific but clearly less sensitive assay. This hierarchy, highlighted in previous work by the co-authors of this study, shows that Viasure and Sansure (based on culture isolates rather than clinical samples) both achieved 100% sensitivity for MTBC, but Viasure clearly outperformed Sansure for NTM (36). Another piece of work from our group has demonstrated the utility of the Viasure assay for detecting NTM in culture-derived samples, identifying a high proportion and diversity of NTM species, as confirmed by MALDI-TOF MS. These findings support its potential as a robust tool for NTM detection, particularly in well-characterized isolates with higher bacterial loads (43). Our results, therefore, extend the existing literature by showing, in a fully clinical, mixed pulmonary/extrapulmonary setting, that Viasure can match Seegene for MTBC detection and may offer a distinct advantage for NTM detection, while Sansure represents a low-sensitivity option that barely outperforms non-molecular traditional methods.

This study has limitations that should be considered when interpreting the results. First, smear microscopy was performed using Ziehl–Neelsen staining, the method routinely used in diagnostic laboratories in Ecuador, rather than fluorescent staining, which has been reported to offer higher sensitivity. Therefore, the performance of smear microscopy in this study may underestimate its potential sensitivity under optimized laboratory conditions. Second, culture was conducted using Lowenstein-Jensen medium, the standard solid medium used in routine clinical practice in the country. However, this medium is known to have lower recovery rates for certain NTM compared to liquid culture systems or Middlebrook-based media, which may have influenced the observed agreement between culture and molecular methods. Third, the distribution of sample types may limit the generalizability of our findings. A high proportion of pulmonary specimens corresponded to sputum, while non-sputum respiratory samples and tissue biopsies were underrepresented. Similarly, extrapulmonary samples accounted for a smaller fraction of the data set and were predominantly composed of urine and fecal samples. As a result, further studies are needed to better characterize the performance of these molecular assays across a broader range of specimen types, particularly tissue-based samples. Importantly, despite these limitations, the diagnostic approaches evaluated in this study reflect real-world laboratory practices in Ecuador, providing relevant insights into the comparative performance of molecular and conventional methods within this setting.

An additional important limitation relates to the characterization of MTBC and NTM. Species-level identification of MTBC and NTM from culture-positive specimens was not performed using biochemical, molecular, or proteomic methods. Instead, MTBC and NTM classification in culture-positive samples was inferred based on concordance with PCR results. Furthermore, most of the molecular assays evaluated (Viasure and Seegene) are designed to detect MTBC and NTM collectively rather than to discriminate among species. As a result, it was not possible to evaluate species-specific diagnostic performance or to determine whether differences in assay sensitivity were associated with particular MTBC and NTM taxa. In addition, the relatively low number of NTM-positive samples in this study further limits the robustness of conclusions regarding NTM detection, as has been previously highlighted in studies in which reduced NTM representation constrains meaningful assessment of assay performance across species (39). Other studies that incorporate well-characterized NTM collections identified by methods such as Sanger sequencing or MALDI-TOF MS are needed to clarify these aspects (44).

Taken together, our data show that Viasure and Seegene provide robust molecular tools for TB diagnosis, with high sensitivity and specificity for MTBC that are broadly in line with previous reports on Anyplex/Seegene performance in clinical specimens (39, 40). In contrast, Sansure behaved as a very less sensitive assay, adding relatively little beyond smear and culture. A key contribution of this work is a clearer separation of NTM performance: while earlier studies hinted at variable NTM sensitivity across Seegene and related assays, our results show that Viasure consistently outperforms both Seegene and Sansure for NTM detection, even when working directly with heterogeneous clinical samples rather than culture isolates. In practical terms, this means that laboratories in medium to high-burden settings that need rapid, direct testing of pulmonary and extrapulmonary specimens would benefit from a high-performing MTBC assay, such as Seegene or Viasure (with Viasure also showing promising performance for NTM detection under these study conditions). On the other hand, the Sansure kit showed limited sensitivity for direct detection of mycobacteria in clinical samples in this study, suggesting that its use should be carefully evaluated.

## Supplementary Material

Reviewer comments

## SUPPLEMENTAL MATERIAL

OPEN PEER REVIEW reviewer-comments.pdf An accounting of the reviewer comments and feedback.

ASM does not own the copyrights to Supplemental Material that may be linked to, or accessed through, an article. The authors have granted ASM a non-exclusive, world-wide license to publish the Supplemental Material files. Please contact the corresponding author directly for reuse.
