# Supplementary material for: Comparative evaluation of three commercial real-time PCR kits for direct detection of Mycobacterium tuberculosis complex and non-tuberculous mycobacteria from pulmonary and extrapulmonary clinical samples: experiences from a low-income and high-burden setting
Source: Microbiol Spectr. 2026 May 18;14(7):e00158-26. doi: 10.1128/spectrum.00158-26 (PMC13340023; doi:10.1128/spectrum.00158-26)
Supplement: Reviewer comments [file reviewer-comments.pdf]

AMERICAN SOCIETY FOR MICROBIOLOGY | Microbiology Spectrum

Comparative evaluation of three commercial Real-Time PCR kits for direct detection of Mycobacterium tuberculosis Complex and Non-Tuberculous Mycobacteria from pulmonary and extrapulmonary clinical samples: experiences from a low income and high burden setting

Ángel Rodríguez-Pazmiño, Elsy Carvajal, Bernardo Castro-Rodriguez, Greta Franco-Sotomayor, Greta Cardenas-Franco, Sandra Uruchima, Solon Orlando, and Miguel Angel Garcia Bereguiain

Corresponding Author(s): Miguel Angel Garcia Bereguiain, Universidad de Las Americas

Review Timeline:

Submission Date:	January 29, 2026
Editorial Decision:	March 18, 2026
Revision Received:	April 27, 2026
Accepted:	May 2, 2026

Editor: Daniel Ortiz

Reviewer(s): The reviewers have opted to remain anonymous.

Transaction Report:

(Note: With the exception of the correction of typographical or spelling errors that could be a source of ambiguity, letters and reports are not edited. The original formatting of letters and referee reports may not be reflected in this compilation.)

DOI: https://doi.org/10.1128/spectrum.00158-26

Re: Spectrum00158-26 (Comparative evaluation of three commercial Real-Time PCR kits for direct detection of Mycobacterium tuberculosis Complex and Non-Tuberculous Mycobacteria from pulmonary and extrapulmonary clinical samples: experiences from a low income and high burden setting)

Dear Dr. Miguel Angel Garcia Bereguiain:

Thank you for the privilege of reviewing your work. Below you will find instructions from the Spectrum editorial office and the reviewer comments.

Please return the manuscript within 60 days; if you cannot complete the modification within this time period, please contact me. If you do not wish to modify the manuscript and prefer to submit it to another journal, notify me immediately so that the manuscript may be formally withdrawn from consideration by Spectrum.

Revision Guidelines
To submit your modified manuscript, log into the submission site at https://spectrum.msubmit.net/cgi-bin/main.plex. Go to Author Tasks and click the appropriate manuscript title to begin. The information you entered when you first submitted the paper will be displayed; update this as necessary. Note the following requirements:

• Upload point-by-point responses to the issues raised by the reviewers in a file named "Response to Reviewers," NOT in your cover letter.
• Upload a compare copy of the manuscript (without figures) as a "Marked-Up Manuscript" file.
• Upload a clean .DOC/.DOCX version of the revised manuscript and remove the previous version.
• Each figure must be uploaded as a separate, editable, high-resolution file (TIFF or EPS preferred), and any multipanel figures must be assembled into one file.
• Any supplemental material intended for posting by ASM should be uploaded with their legends separate from the main manuscript. You can combine all supplemental material into one file (preferred) or split it into a maximum of 10 files with all associated legends included.

For complete guidelines on revision requirements, see our Submission and Review Process webpage. Submission of a paper that does not conform to guidelines may delay acceptance of your manuscript.

Data availability: ASM policy requires that data be available to the public upon online posting of the article, so please verify all links to sequence records, if present, and make sure that each number retrieves the full record of the data. If a new accession number is not linked or a link is broken, provide Spectrum production staff with the correct URL for the record. If the accession numbers for new data are not publicly accessible before the expected online posting of the article, publication may be delayed; please contact production staff (Spectrum@asmusa.org) immediately with the expected release date.

Publication Fees: For information on publication fees and which article types are subject to charges, visit our website. If your manuscript is accepted for publication and any fees apply, you will be contacted separately about payment during the production process; please follow the instructions in that e-mail. Arrangements for payment must be made before your article is published.

ASM Membership: Corresponding authors may join or renew ASM membership to obtain discounts on publication fees. Need to upgrade your membership level? Please contact Customer Service at Service@asmusa.org.

The ASM Journals program strives for constant improvement in our submission and publication process. Please tell us how we can improve your experience by taking this quick Author Survey.

Thank you for submitting your paper to Spectrum.

Sincerely,
Daniel Ortiz
Editor
Microbiology Spectrum

Reviewer #1 (Comments for the Author):

In this manuscript, the authors describe their findings from a retrospective study designed to determine the performance characteristics of three different PCRs, Viasure, Sansure, and Anyplex/Seegene, in detecting Mycobacterium tuberculosis (MTB) complex and nontuberculous mycobacteria (NTM) in suspected cases of pulmonary and extrapulmonary mycobacterial infections. 407 residual clinical specimens (308 pulmonary origin, with 72% sputum; 99 extrapulmonary) from suspected causes of MTB or NTM infections were assessed by AFB smear, AFB culture, and all 3 PCR tests.

The authors undertook 3 different approaches to determine the performance of the 3 PCRs. First, the three PCRs were individually compared against AFB smear and culture. Second, a composite reference standard was used to denote a sample as "positive" if it had a positive result from smear, culture, or any of the 3 PCRs. Third, The Seegene PCR was used as the standard and the Viasure and Sansure performance was compared against this. The summary of the findings was that the Viarasure PCR had the best overall sensitivity and specificity, while the Seegene PCR also performed well but lacked sensitivity for NTM detection, and both of these PCR assays significantly outperformed AFB smear and AFB culture in the detection of MTB and NTM from pulmonary and extrapulmonary specimens. Overall, this is a nice study demonstrating the performance of three different PCRs for the detection of MTB complex and NTMs in pulmonary and extrapulmonary specimens. The manuscript could be further improved for readers by clarifying the concerns and comments listed below prior to publication.

Major Comments
1. The authors do not describe any limitations of this study, but there are certainly limitations that are worth mentioning to the readers for understanding generalized takeaways from the study.
a. AFB smear method was done using Ziehl-Neelson and not fluorescent staining. Fluorescent staining is more sensitive than non-fluorescent staining, so it is possible that there would be more AFB smear-positive specimens that would correlate better with PCR.
b. Lowenstein-Jensen (LJ) agar was used as the culture medium. It is known that LJ media has lower rates of recovery for NTMs compared to Middlebrook media. Additionally, broth culture is superior to solid media for recovery of most mycobacteria, which was not used in this study. For those labs that use Middlebrook and broth systems for AFB cultures, it is possible that they would see better concordance between culture and PCR.
c. 73% of the pulmonary samples tested were sputum, and very few non-sputum specimens were included in this study. It is worth noting that future studies are needed to better understand the performance of any or all of the 3 PCRs with non-sputum specimen types. Similarly, only 24% of specimens tested in this study were of non-pulmonary origin, and most of these were feces and urine. Further studies are needed to better understand the performance of these PCRs in non-pulmonary specimen types, specifically tissue biopsies.

2. It is unclear which NTMs were identified from culture-positive specimens in this study. For culture positive specimens, how was identification performed and what were the findings for MTB complex (within this, how many were M. tuberculosis vs other species?) and NTMs? The methods state that the Viasure kit can distinguish M. tuberculosis from other MTB complex species, but there is no data on how many specimens had M. tuberculosis identified vs other species in the study.
a. The methods also state that the Sansure kit is capable of specifically identifying various NTM species, but no data is presented on this.
b. In general, it would be useful to see a breakdown of performance for the 3 PCRs with different NTMs that were identified in culture. There is no data regarding the specificity of the three PCRs for different NTMs, which it may not be known, but it would be worth commenting on either way. If unknown, future studies would be needed to fully understand the performance of any or all of the PCRs for respective NTMs.
c. Example:
i. Line 436: For NTM, Viasure is the only kit that detects most CRS-positive NTM cases with high specificity, while Seegene and Sansure are extremely specific but miss the majority of NTM infections recognized by the CRS.
ii. Line 477: .....with Viasure clearly strongest for NTM, while Sansure is a very specific but much less sensitive option that behaves close to the traditional methods rather than overcoming their limitations.
iii. Line 508: Viasure can match Seegene for MTBC detection and offers a distinct advantage for NTM, while Sansure represents a low-sensitivity option that barely outperforms non molecular traditional methods.
iv. Without knowing which NTMs were identified in culture-positive specimens or knowing how the tests perform for different NTM isolates, it is impossible to ascertain anything from this data regarding NTM specificity. Line 477 and Lines 508-510 are misleading due to this reason.

Minor Comments
1. For AFB smears in this study, was there any quantification of the positive results (i.e., rare, few, many, etc.)? If not, please explicitly state this. If there was quantification on the AFB smears, it would be useful to see the performance of the three PCRs for different quantities of AFB seen on smear. In general, the sensitivity of molecular testing for AFB is relatively good if AFB smears are positive, and sensitivity is markedly lower on AFB-negative smears.
2. Line 167: "....overview of all comparison across these four schemes" and Line 474 "...the four pieces of analysis...."
a. I believe these should three schemes and pieces, respectively?
3. Line 169: What was the timeframe in which the 407 clinical specimens were collected and tested? It is stated in the sample collection section of the methods that specimens were stored at -20 C until further analysis, but what was the range of time for this across all specimens (days, weeks, months)? This would be helpful for the readers to understand how frozen stability impacted the PCR results, if it had any impact (i.e., did older specimens perform worse than newer specimens, and was this seen across all 3 PCRs equally or did some PCRs detect AFB when others did not on the same specimen?).
4. Line 176: "...and culture, or via real-time PCR in emergency cases".
a. What PCR assay was used for these cases? And where is this information in the tables or results to know which specimens were PCR-positive as part of standard of care testing?
5. Line 438: "...PCRs, Viasure has the highest overall positivity, followed by Seegene, while Sansure is only slightly above

smear and even below culture"
a. It would be useful to add total positivity and percentages for the reader to clearly see the differences.
6. Line 446: "Against smear, Viasure and Seegene show very high sensitivities but low specificities, because they detect many smear-negative samples that smear calls negative"
a. This sentence is confusing. Suggestion: Against smear, Viasure and Seegene show very high sensitivity but low specificity, because they were positive for many smear-negative samples.
7. Line 466: "....., excluding positives with Ct < 35."
a. I believe this should be {greater than or equal to}35.
8. Table 6: I believe that for the row with "Viasure/Smear Microscopy/Total" that the "TP" value is supposed to be 38 (based on TP from pulmonary and extrapulmonary listed in the table) and not 3 as listed.

Reviewer #3 (Comments for the Author):

In this ambitious study, Rodríguez-Pazmiño and colleagues compare the performance of microscopy, bacterial culture and three commercial PCR assays for the detection of mycobacteria from pulmonary and extrapulmonary clinical samples. Although culture was once considered the 'gold standard', this detailed comparison of test results demonstrates that performance characteristics vary with the choice of reference method. The study indicates that, overall, the Viasure and Seegene assays outperform the Sansure test as well as traditional microscopy and culture-based methods.
This work appears to be the latest in a series of studies by these authors focused on evaluation of mycobacterial diagnostics. This may be their first look at the Seegene assay but, in a previous study of mycobacterial isolates, they also found that, for detection of NTM, the Viasure assay was superior to the Sansure test. Curiously, that study (ref 42) is referred to as "the only published evaluation that includes Viasure and Sansure" and not as "previous work by co-authors of our current study". Another relevant study, where co-authors used the Viasure assay for surveillance of mycobacteria in cattle (doi: 10.3389/fvets.2025.1565066), isn't even mentioned.

Major Comments
Methods
The current work involves clinical samples from pulmonary and extrapulmonary sites. However, available product information indicates that the Seegene test is intended for use with sputum, bronchial washings, bacterial colonies (e.g., liquid culture) and fresh tissue whereas the Viasure is designed for detection in mycobacterial culture, clinical isolates of mycobacteria and sputum. According to Table 2, the majority of extrapulmonary samples used in this study represent other specimen types (e.g., feces, urine, gastric aspirates and various fluids). Prior to starting this study, did the authors validate the use of the molecular assays with these other specimen types?
The Methods section clearly describes sample processing for respiratory (esp. sputum) samples. Any modifications used when processing (e.g., sample decontamination, smear preparation, culture, DNA extraction) the extrapulmonary samples should also be included in the Methods.
Although the Methods section does state that "All heat inactivation steps were conducted within the BSL 2+ facility", it would be helpful to describe other biosafety measures. For example, were specimens always handled in a biosafety cabinet (BSC)? Were centrifugation steps performed with sealed rotors that were only opened in a BSC?

Method Comparisons
Scheme 1 used smear microscopy and culture as references. How many of the 407 samples in the study were actually tested by microscopy and culture? Table 1 shows 278 tested by microscopy ("Bacilloscopy") and 147 tested by culture. These are presented as independent tests, but 278 + 147 = 425, not 407.
Although the detailed analysis of the 3 Schemes is an interesting exercise, the composite reference standard (Scheme 2) appears to be the best approach. It is well documented that culture and microscopy are less sensitive than molecular methods. The numerous 'false positive' results associated with Scheme 1 (e.g., Table 6) provide further evidence of that. For Scheme 3, the Seegene assay failed to detect any NTM in extrapulmonary samples whereas the Viasure and Sansure did. However, the Seegene is not marketed to be used with most of the extrapulmonary specimen types used in this study, and thus, shouldn't be used as the reference standard for such samples. Similarity, the Seegene is marketed as a 'qualitative' assay. As long as the internal controls are valid, any positive should be reported. Or, if the exclusion of positive samples with Ct {greater than or equal to} 35 is part of the "manufacturer defined threshold and cutoff values", that should be clearly stated in the Methods
Because the extra analyses don't actually change the results of the study, consider removing Tables 3-6 and Tables 11-13 from the manuscript. Focusing exclusively on the CRS analysis would make the narrative simpler and more straightforward. A brief statement should be sufficient to justify the choice of a composite reference standard. Alternately, the data associated with Schemes 1 & 3 may be suitable as Supplementary Data. A new Table that lists all of the results for the 407 samples might also be useful as Supplementary Data.

Additional comments
One comparison that does not appear in the current manuscript is positivity by clinical sample type. That information could be included by adding columns to Table 2
The reference numbering should be reviewed. Several of the early references don't correspond to what is in the text. For example, lines 103-108 describe NTM infections but supporting ref 5 refers to drug resistance in MTBC and ref 6 is about

diagnostics, not disease presentation.
In the manuscript, the culture results are presented as 'mycobacteria'. However, it should be possible to classify positive cultures to the species level. How many positive cultures were NTM? Which NTM species were present? Which species were - or were not - consistently detected by the molecular assays?

Manuscript Number: JCTUBE-D-25-00253

Title: Tuberculous lymphadenitis, extrapulmonary tuberculosis, GeneXpert Ultra, molecular diagnostics, Saudi Arabia

Recommendation: Major revisions

Reviewer Comments to the Authors:

In this manuscript, the authors describe their findings from a retrospective study for the detection of *Mycobacterium tuberculosis* (MTB) complex in suspected cases of tuberculous lymphadenitis using the Cepheid GeneXpert MTB/RIF Ultra PCR assay, Cepheid GeneXpert MTB/RIF PCR assay, and BD ProbeTec PCR. Molecular testing was performed on lymph node biopsies that had varying degrees of routine testing performed on them (histopathology, AFB culture, AFB smear), and comparison of the different PCR assays against AFB cultures that were performed on respective specimens to determine clinical sensitivities for the detection of MTB complex.

Eighty-five cases of TB lymphadenitis were used in this study, most of which had core needle biopsies as the specimen collection method. AFB culture-positivity and AFB smear-positivity of specimens that were tested by respective methods were low (14.81 and 43.59%, respectively), which is expected given the low TB-endemicity of the region in which this study was performed. Of the specimens that had PCR testing performed, various sample sizes and sensitivities were reported: Xpert Ultra (26/63; 41% positivity), MTB/RIF (6/19; 31% positivity), and ProbeTec (1/3; 33.3%positivity).

Of the specimens that were culture-positive (N = 17), 85% (12/14) were positive by Xpert Ultra, 100% (3/3) were positive by MTB/RIF, and no positive culture specimens were tested by ProbeTec assay. Of the 22 culture-negative specimens that were tested by a PCR method, 16% (3/18) were positive by Xpert Ultra and zero were positive by MTB/RIF (N = 3) or ProbeTec (N = 1).

Overall, this study demonstrates that the GeneXpert MTB/RIF Ultra assay is capable of detecting MTB complex in lymph node biopsies from suspected cases of TB lymphadenitis and likely performs similar to the GeneXpert MTB/RIF assay. However, culture remains the gold-standard for MTB diagnosis and molecular testing should be performed as a more rapid, adjunctive diagnostic to culture. Future studies are needed to investigate true head-to-head performance of PCR assays in this patient population to better understand performance characteristics in cases of suspected MTB lymphadenitis in low TB-endemicity areas. The study is worthy of publishing, but the manuscript could benefit from additional information prior to publication regarding methods for how the three PCR assays were performed (specimen processing, input volumes, etc.), whether all lymph nodes biopsied from a given patient were tested by histopathology, culture, AFB smear, and PCR, and further clarification that due to the lack of a head-to-head comparison by testing the same specimens on all three PCR assays, there is insufficient data to compare the clinical sensitivity of the three assays to one another. The comparison of the

individual PCR assays to culture is appropriate, although it should be further highlighted that due to the variable sample sizes between the three PCR assays, these results may not be generalizable.

Major Comments

1. **1.** There are no methods descriptions for how specimens were processed and run on the Xpert MTB/RIF ultra. No methods described for the Xpert MTB/RIF or the ProbeTec PCRs.
2. **2.** It is unclear if all lymph nodes involved were tested by culture, smear, and PCR. Please clarify for patients who had multiple lymph nodes involved, was all testing performed on each specimen. If not, there should be details provided about how many lymph nodes were tested and by which method.
   - Example: If one patient had 4 lymph nodes biopsied, how many had histopathology, how many had cultures, how many had AFB smears, and how many had PCR?
   - Were there any cases where PCR was performed on a lymph node that was culture-negative, but a different lymph node from the same patient was culture-positive that did not have PCR testing?
   - If there were cases where not all testing was performed on every lymph node biopsied, were these excluded from the data set?
3. **3.** Conclusion: The following statement seems misleading based on the lack of head-to-head comparison of all 3 PCRs on the same specimens and the large differences in total number of specimens tested by each PCR:
   - "While GeneXpert Ultra demonstrated higher sensitivity than its predecessor and other molecular platforms, its performance remained suboptimal".

   Based on the data presented and how the study was carried out, we don't really know if the Xpert Ultra is more clinically sensitive than either of the other two PCRs for MTB lymphadenitis in this study. A suggestion would be to state that the GeneXpert Ultra detected more cases (could just be a result of increased sample size) of MTB than the other two PCRs, which is factually true based on the data presented, but does not make a claim that the sensitivity is higher, which would have required a head-to-head comparison of all 3 PCRs tested on the same specimens. The authors reference two manuscripts in the fourth paragraph of the Introduction (reference 13 and 14), both of which performed head-to-head comparisons of the molecular methods used in the respective studies. The authors do acknowledge this as a limitation in the discussion but still reference the Xpert Ultra as being more sensitive throughout the discussion, which has not been demonstrated in this study.

Minor Comments

1. Introduction; first line: Please italicize "Mycobacterium tuberculosis".

2. Throughout the document, the use of "Xpert Ultra" and "Ultra" are used interchangeably to describe the GeneXpert MTB/RIF Ultra assay. Please unify this throughout the document to one of the two.

3. Last paragraph in the Results section: Were the specimens that were culture-positive but Xpert Ultra negative tested by either of the other two PCRs? And were the 3-culture negative cases that were positive by the Xpert Ultra, were these tested by either of the other 2 PCRs? If "yes" to these questions, please comment on that within the manuscript.

4. How many of the lymph nodes that were tested had pathology performed on them with acid-fast staining done on the FFPE(Table 2)? If AFB staining was done on the FFPE tissue, it would be interesting to see how many of these also had fresh tissue tested by PCR and what the FFPE AFB staining result was (i.e., if AFB were seen on FFPE, how many of these were PCR-positive/negative? If AFB-negative on FFPE, how many were PCR-positive/negative?)

    a. Often, if AFB are seen on FFPE, the sensitivity of PCR is quite high. If AFB are not seen, sensitivity is often low.

5. Discussion, paragraph 8: The authors list the LoD of the Xpert MTB/RIF Ultra assay. It would be helpful to list the LoDs in the methods or in the results of all 3 PCR assays that were used, so that readers have a sense of differences in analytical sensitivity of the assays used.

Dear Editor,

Please, see below our point-by-point response to every comment of the 3 reviewers.

Also, all changes in the manuscript are highlighted in red for clarity in one of the uploaded files.

We believe we have improved our manuscript substantially so now we hope it is suitable for publication.

Best regards

Miguel Angel Garcia Bereguiain

Reviewer #1

In this manuscript, the authors describe their findings from a retrospective study designed to determine the performance characteristics of three different PCRs, Viasure, Sansure, and Anyplex/Seegene, in detecting Mycobacterium tuberculosis (MTB) complex and nontuberculous mycobacteria (NTM) in suspected cases of pulmonary and extrapulmonary mycobacterial infections. 407 residual clinical specimens (308 pulmonary origin, with 72% sputum; 99 extrapulmonary) from suspected causes of MTB or NTM infections were assessed by AFB smear, AFB culture, and all 3 PCR tests.

The authors undertook 3 different approaches to determine the performance of the 3 PCRs. First, the three PCRs were individually compared against AFB smear and culture. Second, a composite reference standard was used to denote a sample as "positive" if it had a positive result from smear, culture, or any of the 3 PCRs. Third, The Seegene PCR was used as the standard and the Viasure and Sansure performance was compared against this. The summary of the findings was that the Viarasure PCR had the best overall sensitivity and specificity, while the Seegene PCR also performed well but lacked sensitivity for NTM detection, and both of these PCR assays significantly outperformed AFB smear and AFB culture in the detection of MTB and NTM from pulmonary and extrapulmonary specimens. Overall, this is a nice study demonstrating the performance of three different PCRs for the detection of MTB complex and NTMs in pulmonary and extrapulmonary specimens. The manuscript could be further improved for readers by clarifying the concerns and comments listed below prior to publication.

Answer: Thanks for the positive feedback. We have improved the manuscript following your recommendations.

Major Comments

1. The authors do not describe any limitations of this study, but there are certainly limitations that are worth mentioning to the readers for understanding generalized takeaways from the study.

a. AFB smear method was done using Ziehl-Neelson and not fluorescent staining. Fluorescent staining is more sensitive than non-fluorescent staining, so it is possible that there would be more AFB smear-positive specimens that would correlate better with PCR.

Answer: Agree. The AFB method described is the one used for routine diagnosis in Ecuador. This is listed now as a limitation of the study.

b. Lowenstein-Jensen (LJ) agar was used as the culture medium. It is known that LJ media has lower rates of recovery for NTMs compared to Middlebrook media. Additionally, broth culture is superior to solid media for recovery of most mycobacteria, which was not used in this study. For those labs that use Middlebrook and broth systems for AFB cultures, it is possible that they would see better concordance between culture and PCR.

Answer: Agree. The culture method described is the one used for routine diagnosis in Ecuador. This is listed now as a limitation of the study.

c. 73% of the pulmonary samples tested were sputum, and very few non-sputum specimens were included in this study. It is worth noting that future studies are needed to better understand the performance of any or all of the 3 PCRs with non-sputum specimen types. Similarly, only 24% of specimens tested in this study were of non-pulmonary origin, and most of these were feces and urine. Further studies are needed to better understand the performance of these PCRs in non-pulmonary specimen types, specifically tissue biopsies.

Answer: Agree. The sample collection included in our study has limitations that we now acknowledge.

2. It is unclear which NTMs were identified from culture-positive specimens in this study. For culture positive specimens, how was identification performed and what were the findings for MTB complex (within this, how many were M. tuberculosis vs other species?) and NTMs? The methods state that the Viasure kit can distinguish M. tuberculosis from other MTB complex species, but there is no data on how many specimens had M. tuberculosis identified vs other species in the study.

Answer: NTM species identification was not done at culture level by biochemical methods or similar. For the sake of comparison, when a sample was identified as NTM by PCR and was also positive in culture, it was considered NTM by culture.

a. The methods also state that the Sansure kit is capable of specifically identifying various NTM species, but no data is presented on this.

Answer: The performance of Sansure for NTM was so poor that it made no sense to analyze further those results in terms of NTM species. Sansure works well for NTM species determination when NTM culture samples are used, as we have already published.

b. In general, it would be useful to see a breakdown of performance for the 3 PCRs with different NTMs that were identified in culture. There is no data regarding the specificity of the three PCRs for different NTMs, which it may not be known, but it would be worth commenting on either way. If unknown, future studies would be needed to fully understand the performance of any or all of the PCRs for respective NTMs.

Answer: A main limitation of the study is the reduced number of NTMs included, and also that no species identification is done at the culture level. PCR kits like Seegene and Viasure do not discriminate among NTM species and only identify NTMs as a whole group, rather than MTBC. Further studies, including the collection of NTMs identified at the species level using Sanger sequencing, MALDI-TOF, or similar methods, are now suggested as future directions following the description of limitations.

c. Example:
i. Line 436: For NTM, Viasure is the only kit that detects most CRS-positive NTM cases with high specificity, while Seegene and Sansure are extremely specific but miss the majority of NTM infections recognized by the CRS.
ii. Line 477: .....with Viasure clearly strongest for NTM, while Sansure is a very specific but much less sensitive option that behaves close to the traditional methods rather than overcoming their limitations.
iii. Line 508: Viasure can match Seegene for MTBC detection and offers a distinct advantage for NTM, while Sansure represents a low-sensitivity option that barely outperforms non molecular traditional methods.
iv. Without knowing which NTMs were identified in culture-positive specimens or knowing how the tests perform for different NTM isolates, it is impossible to ascertain anything from this data regarding NTM specificity. Line 477 and Lines 508-510 are misleading due to this reason.

Answer: Thanks for pointing this out. As we stated previously, we could not identify any NTM species in our study. We have addressed NTM specificity as a group by opposing MTBC, because this is how the Seegene and Viasure kits work. Nevertheless, all the lines of the text indicated by the reviewers have been revised and rephrased for clarity.

Minor Comments
1. For AFB smears in this study, was there any quantification of the positive results (i.e., rare, few, many, etc.)? If not, please explicitly state this. If there was quantification on the AFB smears, it would be useful to see the performance of the three PCRs for different quantities of AFB seen on smear. In general, the sensitivity of molecular testing for AFB is relatively good if AFB smears are positive, and sensitivity is markedly lower on AFB-negative smears.

Answer: Unfortunately, the AFB smears results was only positive of negative. This is now clearly stated in the methods.

2. Line 167: "....overview of all comparison across these four schemes" and Line 474 "...the four pieces of analysis...."
a. I believe these should three schemes and pieces, respectively?

Answer: corrected.

3. Line 169: What was the timeframe in which the 407 clinical specimens were collected and tested? It is stated in the sample collection section of the methods that specimens were stored at -20 C until further analysis, but what was the range of time for this across all specimens (days, weeks, months)? This would be helpful for the readers to understand how frozen stability impacted the PCR results, if it had any impact (i.e., did older specimens perform worse than newer specimens, and was this seen across all 3 PCRs equally or did some PCRs detect AFB when others did not on the same specimen?).

Answer: Thanks for pointing this out. The samples were collected during a 2-month period. All samples were processed for PCR kits simultaneously, so no more than one thawing cycle and no more than two months of -20 °C storage were applied to any sample. This is detailed now in the methods.

4. Line 176: "...and culture, or via real-time PCR in emergency cases".
a. What PCR assay was used for these cases? And where is this information in the tables or results to know which specimens were PCR-positive as part of standard of care testing?

Answer: In fact, PCR testing in emergency situations is performed using GeneXpert tests. This information, provided for reference purposes, is based on a personal communication to the authors, for which we have no supporting data. Accordingly, we have replaced "real-time PCR" with "GeneXpert assays" and added a note indicating that this is based on a personal communication.

5. Line 438: "...PCRs, Viasure has the highest overall positivity, followed by Seegene, while Sansure is only slightly above smear and even below culture"
a. It would be useful to add total positivity and percentages for the reader to clearly see the differences.

Answer: corrected.

6. Line 446: "Against smear, Viasure and Seegene show very high sensitivities but low specificities, because they detect many smear-negative samples that smear calls negative"
a. This sentence is confusing. Suggestion: Against smear, Viasure and Seegene show very high sensitivity but low specificity, because they were positive for many smear-negative samples.

Answer: corrected.

7. Line 466: "....., excluding positives with Ct < 35."
a. I believe this should be {greater than or equal to}35.

Answer: corrected.

8. Table 6: I believe that for the row with "Viasure/Smear Microscopy/Total" that the "TP" value is supposed to be 38 (based on TP from pulmonary and extrapulmonary listed in the table) and not 3 as listed.

Answer: corrected.

Reviewer #3

In this ambitious study, Rodríguez-Pazmiño and colleagues compare the performance of microscopy, bacterial culture and three commercial PCR assays for the detection of mycobacteria from pulmonary and extrapulmonary clinical samples. Although culture was once considered the 'gold standard', this detailed comparison of test results demonstrates that performance characteristics vary with the choice of reference method. The study indicates that, overall, the Viasure and Seegene assays outperform the Sansure test as well as traditional microscopy and culture-based methods.
This work appears to be the latest in a series of studies by these authors focused on evaluation of mycobacterial diagnostics. This may be their first look at the Seegene assay but, in a previous study of mycobacterial isolates, they also found that, for detection of NTM, the Viasure assay was superior to the Sansure test. Curiously, that study (ref 42) is referred to as "the only published evaluation that includes Viasure and Sansure" and not as "previous work by co-authors of our current study". Another relevant study, where co-authors used the Viasure assay for surveillance of mycobacteria in cattle (doi: 10.3389/fvets.2025.1565066), isn't even mentioned.

Answer: Thanks for the positive feedback. We have corrected the way to refer to that reference, and we have now included our publication on cattle around lines 496 to 500.

Major Comments
Methods
The current work involves clinical samples from pulmonary and extrapulmonary sites. However, available product information indicates that the Seegene test is intended for use with sputum, bronchial washings, bacterial colonies (e.g., liquid culture) and fresh tissue whereas the Viasure is designed for detection in mycobacterial culture, clinical isolates of mycobacteria and sputum. According to Table 2, the majority of extrapulmonary samples used in this study represent other

specimen types (e.g., feces, urine, gastric aspirates and various fluids). Prior to starting this study, did the authors validate the use of the molecular assays with these other specimen types?

Answer: Thanks for pointing this out. We did not perform that validation, but we asked the manufacturers, and they said that the list of potential samples included in the instructions for each kit refers to samples that are internally validated, without implying that other samples cannot be used. Moreover, the kits include an internal control to ensure that PCR inhibition does not mask false-negative results. This info is now detailed in the methods. This info was included in lines 272 to 281, in the methods section.

The Methods section clearly describes sample processing for respiratory (esp. sputum) samples. Any modifications used when processing (e.g., sample decontamination, smear preparation, culture, DNA extraction) the extrapulmonary samples should also be included in the Methods.
Although the Methods section does state that "All heat inactivation steps were conducted within the BSL 2+ facility", it would be helpful to describe other biosafety measures. For example, were specimens always handled in a biosafety cabinet (BSC)? Were centrifugation steps performed with sealed rotors that were only opened in a BSC?

Answer: Thanks for pointing this out. This info was added to the methods in lines 211-216.

Method Comparisons
Scheme 1 used smear microscopy and culture as references. How many of the 407 samples in the study were actually tested by microscopy and culture? Table 1 shows 278 tested by microscopy ("Bacilloscopy") and 147 tested by culture. These are presented as independent tests, but 278 + 147 = 425, not 407.

Answer: This info was clarified in lines 164-168. There was an overlap of 18 samples between the results of both methods.

Although the detailed analysis of the 3 Schemes is an interesting exercise, the composite reference standard (Scheme 2) appears to be the best approach. It is well documented that culture and microscopy are less sensitive than molecular methods. The numerous 'false positive' results associated with Scheme 1 (e.g., Table 6) provide further evidence of that. For Scheme 3, the Seegene assay failed to detect any NTM in extrapulmonary samples whereas the Viasure and Sansure did. However, the Seegene is not marketed to be used with most of the extrapulmonary specimen types used in this study, and thus, shouldn't be used as the reference standard for such samples. Similarity, the Seegene is marketed as a 'qualitative' assay. As long as the internal controls are valid, any positive should be reported. Or, if the exclusion of positive samples with Ct {greater than or equal to} 35 is part of the "manufacturer defined threshold and cutoff values", that should be clearly stated in the Methods.

Answer: Thank you for your observations. The use of the cut-off was applied to improve the robustness of comparisons between assays under low-bacterial-load conditions; it is not based on manufacturer-defined reporting criteria. This was clarified in lines 154-158. Additionally, a

justification for using the Seegene assay as a reference comparator was provided in lines 485-493.

Because the extra analyses don't actually change the results of the study, consider removing Tables 3-6 and Tables 11-13 from the manuscript. Focusing exclusively on the CRS analysis would make the narrative simpler and more straightforward. A brief statement should be sufficient to justify the choice of a composite reference standard. Alternately, the data associated with Schemes 1 & 3 may be suitable as Supplementary Data. A new Table that lists all of the results for the 407 samples might also be useful as Supplementary Data.

Answer: We thank you for this valuable suggestion and agree that focusing exclusively on the CRS would simplify the narrative. However, we would prefer to retain the three analytical schemes, as each addresses a distinct and complementary aspect of the study. Scheme 1 reflects the conventional diagnostic framework, in which smear microscopy and culture are used as reference standards. Scheme 2, based on the CRS, allows us to account for the known limitations in sensitivity of these traditional methods and to better contextualize the apparent false-positive results observed with molecular assays. Finally, Scheme 3 provides an additional layer of comparison between molecular methods, illustrating their performance under conditions of reduced analytical variability (e.g., excluding high Ct values). Taken together, these approaches offer a more comprehensive evaluation of assay performance in a real-world diagnostic setting.

Additional comments
One comparison that does not appear in the current manuscript is positivity by clinical sample type. That information could be included by adding columns to Table 2

Answer: We thank you for this suggestion. While we agree that positivity by specimen type is clinically relevant, most categories in our dataset included very small numbers of samples, limiting the interpretability of such comparisons. However, we have included this information in the Supplementary Material. Our main focus is the analysis of positivity rates for pulmonary, extrapulmonary, and total specimens, which provide more robust and meaningful comparisons for the study that we are proposing (described in Table 3).

The reference numbering should be reviewed. Several of the early references don't correspond to what is in the text. For example, lines 103-108 describe NTM infections but supporting ref 5 refers to drug resistance in MTBC and ref 6 is about diagnostics, not disease presentation.

Answer: Done. Those references are fixed and updated.

In the manuscript, the culture results are presented as 'mycobacteria'. However, it should be possible to classify positive cultures to the species level. How many positive cultures were NTM? Which NTM species were present? Which species were - or were not - consistently detected by the molecular assays?

Answer: Done. In our study, culture results were reported as 'mycobacteria', as species-level identification (including differentiation between MTBC and NTM) was not systematically performed within the routine diagnostic workflow at INSPI. Therefore, we are unable to determine the proportion or identity of MTBC and NTM among culture-positive samples. We have now acknowledged this limitation in lines 557 - 565.

Re: Spectrum00158-26R1 (Comparative evaluation of three commercial Real-Time PCR kits for direct detection of Mycobacterium tuberculosis Complex and Non-Tuberculous Mycobacteria from pulmonary and extrapulmonary clinical samples: experiences from a low income and high burden setting)

Dear Dr. Miguel Angel Garcia Bereguiain:

Your manuscript has been accepted, and I am forwarding it to the ASM production staff for publication. Your paper will first be checked to make sure all elements meet the technical requirements. ASM staff will contact you if anything needs to be revised before copyediting and production can begin. Otherwise, you will be notified when your proofs are ready to be viewed.

Data Availability: ASM policy requires that data be available to the public upon online posting of the article, so please verify all links to sequence records, if present, and make sure that each number retrieves the full record of the data. If a new accession number is not linked or a link is broken, provide production staff with the correct URL for the record. If the accession numbers for new data are not publicly accessible before the expected online posting of the article, publication may be delayed; please contact ASM production staff immediately with the expected release date.

Publication Fees: For information on publication fees and which article types have charges, please visit our website. We have partnered with Copyright Clearance Center (CCC) to collect author charges. If fees apply to your paper, you will receive a message from no-reply@copyright.com with further instructions. For questions related to paying charges through RightsLink, please contact CCC at ASM_Support@copyright.com or toll free at +1-877-622-5543. CCC makes every attempt to respond to all emails within 24 hours.

ASM Membership: Corresponding authors may join or renew ASM membership to obtain discounts on publication fees. Need to upgrade your membership level? Please contact Customer Service at Service@asmusa.org.

Embargo Policy: A press release may be issued as soon as the manuscript is posted on the Spectrum Latest Articles webpage. The corresponding author will receive an email with the subject line "ASM Journals Author Services Notification" when the article is available online.

The ASM Journals program strives for constant improvement in our submission and publication process. Please tell us how we can improve your experience by taking this quick Author Survey.

Thank you for submitting your paper to Spectrum.

Sincerely,
Daniel Ortiz
Editor
Microbiology Spectrum
